# Advancing Organoid Engineering for Tissue Regeneration and Biofunctional Reconstruction

**DOI:** 10.34133/bmr.0016

**Published:** 2024-04-15

**Authors:** Hairong Jin, Zengqi Xue, Jinnv Liu, Binbin Ma, Jianfeng Yang, Lanjie Lei

**Affiliations:** ^1^ Institute of Translational Medicine, Zhejiang Shuren University, Hangzhou 310015, China.; ^2^ The Third Affiliated Hospital of Wenzhou Medical University, Wenzhou 325200, China.; ^3^ Ningxia Medical University, Ningxia 750004, China.; ^4^ Department of Biology, The Johns Hopkins University, Baltimore, MD 21218, USA.

## Abstract

Tissue damage and functional abnormalities in organs have become a considerable clinical challenge. Organoids are often applied as disease models and in drug discovery and screening. Indeed, several studies have shown that organoids are an important strategy for achieving tissue repair and biofunction reconstruction. In contrast to established stem cell therapies, organoids have high clinical relevance. However, conventional approaches have limited the application of organoids in clinical regenerative medicine. Engineered organoids might have the capacity to overcome these challenges. Bioengineering—a multidisciplinary field that applies engineering principles to biomedicine—has bridged the gap between engineering and medicine to promote human health. More specifically, bioengineering principles have been applied to organoids to accelerate their clinical translation. In this review, beginning with the basic concepts of organoids, we describe strategies for cultivating engineered organoids and discuss the multiple engineering modes to create conditions for breakthroughs in organoid research. Subsequently, studies on the application of engineered organoids in biofunction reconstruction and tissue repair are presented. Finally, we highlight the limitations and challenges hindering the utilization of engineered organoids in clinical applications. Future research will focus on cultivating engineered organoids using advanced bioengineering tools for personalized tissue repair and biofunction reconstruction.

## Introduction

Tissue damage and abnormal functioning resulting from endogenous (e.g., genetic diseases [1,2] and tumors [3,4]) or exogenous (e.g., infections [5–8] and traumas [9,10]) factors not only impose considerable physical, psychological, and economic burdens on individuals and their families but also place enormous pressure on public healthcare systems. Human organs typically have limited regenerative capabilities, hindering the achievement of tissue repair and functional organ reconstruction. Consequently, various organ-based diseases were historically managed using pharmaceuticals or surgical interventions to achieve organ reconstruction or regeneration. However, these approaches often lead to infection and recurrence with limited success [11]. Meanwhile, autologous organ transplantation may lead to donor site damage, while allogeneic or prosthetic transplantation can cause immune rejection or inflammation. Thus, traditional methods no longer meet the needs of patients seeking pain-minimizing approaches to tissue repair and restoration of normal physiological function. Fortunately, scholars’ rapidly evolving understanding of organ damage and repair has paved the way for regenerative medicine [12–15].

After decades of research, techniques have been established to regulate the behavior of cells at the molecular level. For example, cells can self-organize along a specific spectrum [16]. Moreover, since isolated stem cells can differentiate into desired mature cells to repair organs [17], they have been applied to in vitro organ reconstruction. Indeed, the development of bioengineering and biomaterials has propelled the advancement of organoid technology [18–21]. The culture of organoids is consuming, and the construction process is complex and requires more cutting-edge bioengineering techniques and longer cycle times; it signifies the dramatic changes that organoid technology may bring to the field of biology. Undeniably, compared with traditional two-dimensional (2D) cell cultures, organoids resemble primary tissues in both composition and structure and contain a population of stem cells that are genome-stable and self-renewing, generating fully differentiated progeny (including all major cell lineages) at a frequency similar to that of the original tissue. Organoids derived from primary tissues also lack mesenchyme, which provides a system for studying specific tissues independent of the local microenvironment. Furthermore, we can modulate the targeted development of organoids for clinical applications by changing parameters at the molecular, cellular, tissue, and systemic levels, which is unmatched by stem cell therapy [22]. In contrast to the limitations of direct stem cell therapy (e.g., low clinical relevance, high heterogeneity, immune rejection, tumorigenicity, and inability to reproduce organ function), organoids from specific tissues can produce the structural features of human organs while preserving their physiological functions [23]. Hence, organoid technology has made important strides in the study of typical physiological structures, biological behaviors [24], disease occurrence [25,26], and drug development [27,28], holding promise for replacing animal models in medical research [29,30].

Recent studies have shown that organoids are important for achieving tissue repair and biofunctional reconstruction. However, despite their potential, organoids have not yet gained widespread use in clinical regenerative medicine research due to the limitations of traditional strategies and models. These challenges might be addressed by engineering organoids. Bioengineering is a multidisciplinary field that applies engineering principles and design to promote human health [31,32]. Applying bioengineering principles to organoids can enhance reproducibility, ensure experimental controllability, improve culture efficiency, optimize the tissue structure and physiological function of organoids, and pave the way for the clinical translation of organoids. Engineered organoids can be used to treat injuries caused by most factors, including various disease types affecting the brain [33], skin [34], bone and cartilage [35,36], liver [37], and endocrine glands [38]. Moreover, organoid engineering approaches have been successfully applied to organoid construction to achieve tissue repair and biofunction reconstruction. Extensive exploration has been conducted on organoid culture models and engineering design methods for regenerative medicine. However, organoids face several difficulties and challenges in terms of cultivation, function, and ethics, and a great deal of basic research support is warranted prior to clinical translation.

In this review, we discuss the basic concepts of engineered organoids and provide a brief guide to the basic process of culturing organoids from different cellular sources, provide an overview of diverse culture protocols developed to engineering organoids, and outline the research framework for engineering organoids. In addition, clinical translational results of the application of engineered organoids to biofunctional reconstruction and tissue repair are presented. Finally, we address the limitations and challenges associated with organoid engineering in tissue repair and functional reconstruction to guide future research directions in this field and inspire novel research designs (Fig. S1).

## Engineering Organoids

### History: From organoid to engineered organoid

Organoids are derived from embryonic stem cells (ESCs), induced pluripotent stem cells (iPSCs), or primitive tissues. Their 3D structure has a unique capacity for self-renewal and self-organization, resembles a multicellular organ, and displays in vivo function. The origin of organoids dates back to 1907 when a biologist, Wilson, demonstrated that sponge cells could self-organize into new sponges with normal function [39]. Subsequently, in 1965, organoids were defined as having abnormal cell growth or intracellular structures in 3D cell cultures. Long-term culture of human cells was first described by Rheinwatd and Green in 1975 [40,41], whereas in 1981, ESCs were first isolated from mouse embryos [42]. In 1998, human ESCs (hESCs) were first isolated from human blastocysts [43]. In 2006, iPSCs were first prepared from mouse somatic cells [44], and in 2008, Eiraku et al. [45] obtained cortical tissues from ESCs. In 2009, Sato et al. [46] demonstrated that crypt fuzzy villi-like organoids could be grown using Lgr5^+^ intestinal stem cells with self-organizing and differentiating capabilities. Currently, 3D cultured organoids can be constructed in vitro from most tissues [47]. During the long historical dimension of organoids [48,49], many cultivation methods have been developed, and organoid engineering technology has attracted considerable attention. The molecular, cellular, and niche-level engineering of organoids holds great promise for tailored organoid culture and maturation. These innovations are poised to increase organoid production, improve reproducibility, and facilitate the creation of more sophisticated organoid culture models.

### Origins of organoid

Organoids can be categorized as adult stem cell (ASC)-origin or PSC-origin based on their cellular origin. Organoids of different cellular origins have commonalities and unique characteristics, which have implications for their culture and application.

#### ASC-derived organoids

ASCs are fundamental for maintaining tissue homeostasis, growth, and regeneration. Upon activation by specific signals, they can proliferate and differentiate into functional cells, crucial for tissue renewal and repair in response to injury or disease [50]. Using a more streamlined protocol, ASC-derived organoids can be directly obtained from human tissue [16,51]. Moreover, the use of human ASCs is ethically noncontroversial, as they are derived from adult tissues rather than embryos. However, the limited potency of ASCs restricts the associated organoids to a single epithelial cell type [18]. Despite this limitation, compared with PSC-derived organoids, ASC-derived organoids closely resemble mature adult tissues, making them better suited for modeling adult tissue repair, and can be amplified in vitro for long periods while maintaining genetic stability [16]. These organoids reproduce the structure and function of tissues and organs through stem cell-based approaches [52]. However, for tissue-specific organoid cells with stem cell properties, such as redifferentiation potential, that cannot be obtained from adult organs (e.g., the heart), ASC-derived organoids may not be feasible.

#### Pluripotent stem cell-derived organoids

Both ESCs and iPSCs can be used to culture organoids. ESCs, originating from the endoderm, can differentiate into all three germ cell layers and exhibit unlimited self-proliferation and renewal capacity, as well as multi-directional differentiation potential [53]. For example, Zhang et al. [54] constructed spheroids using human PSCs (hPSCs) and reconstructed neural tube structures in vitro.

Similar to ESCs, iPSCs can differentiate into three germline cell types [55]. To date, many researchers have devised various protocols for inducing patient-derived somatic cells to generate organoids. Re-editing somatic cells into iPSC using substances such as small-molecule compounds or transcription factors. However, when the endpoint of differentiation is reached, iPSC-derived organoids often fail to continue expanding. At the same time, the use of iPSCs is associated with the risk of tumorigenicity after in vivo transplantation. Due to the pluripotency of iPSCs, the cellular composition of iPSC-derived organoids is more complex [16], making them suitable for studying early organogenesis [18]. For example, Zheng et al. [33] evaluated midbrain organoids cultured from human iPSCs at different times to establish optimal culture patterns. Ultimately, they functionally integrated cultures into striatal circuits and treated Parkinson’s disease (PD) in mice. Besides, the authors proposed a solution to the problem of tumorigenicity that iPSC may cause. Hence, iPSCs can be used to produce organs that cannot be regenerated in the adult body.

In conclusion, both ASC-derived and PSC-derived organoids are valuable tools for tissue repair and biofunction reconstruction; however, they have different advantages and disadvantages that must be considered when applying them. These will be discussed in the “Biological characteristics” section.

#### Cultivate organoids according to needs

The starting cell population is essential for any organoid since it affects not only the function of the tissue they are intended to mimic but also the heterogeneity and variability of the structure. ASC- and PSC-derived organoids can be viewed as complementary systems encompassing developmental and adult tissue states, respectively. ASC-derived organoids allow for the study of processes occurring during tissue regeneration in adults; however, they are present only in a limited number of organs (with regenerative capacity), and all ASC-derived organoid types represent only the epithelial portion of an organ. For example, ASC may be a better source of cells when constructing organoids to repair damaged skin. PSCs, however, are more reflective of organ formation from embryonic development and hence are not limited in the types of organs they can generate. For example, when developing a heart from cells (or even in the future to completely replicate the structure and function of the heart in vivo), PSCs are a better choice. Therefore, as organoids are applied to tissue repair and biofunction reconstruction, we must choose the cell source according to specific research directions or clinical needs.

After identifying the cell source, to establish an ASC-derived organoid, the corresponding local tissue-resident stem cells must be obtained using an optimized dissociation method and embedded in a 3D matrix that mimics the stem cell niche. The starting cell population is usually obtained from adult or fetal tissue biopsy samples. The most commonly used tissue dissociation method is enzymatic digestion, which lyses extracellular matrix (ECM) [56]. Differences in the efficacy of the enzymatic process and the composition of the enzyme mixture occur depending on the tissue type [57]. To remove excess DNA released by necrotic cells, deoxyribonuclease (DNase) can be added in some cases [58]. Depending on the tissue type, the fragments can be incubated with enzymes such as elastase to generate a single-cell suspension, which is then inoculated into Matrigel. The enzymatic method of dissociation may affect the status of the recovered cells as it may require an extended duration of the enzymatic mixture to dissociate most ASCs. Tissue dissociation can also be achieved mechanically. Mechanical dissociation is faster and more cost-effective, although the cell yield and viability are inconsistent [57].

Combining mechanical and enzymatic dissociations produces better cell yields. After tissue dissociation, known biomarkers (e.g., tissue-specific stem cell markers) or physical characteristics are used to identify and collect ASCs for organoid development [56,59]. Depending on multiple parameters (e.g., cell surface marker expression, shape, and size), magnetically activated cell sorting or fluorescently activated cell sorting can be used to isolate cells [56,57]. Laser capture microdissection and manual cell selection are alternatives [56]. As starting cells, PSC lines have been established and fully characterized for PSC-derived organoids. Prior to this, however, there had been some differences in the way ESC and iPSC were obtained. ESC has been isolated from early embryos (prior to the proto-gastrula stage) or primitive gonads, whereas iPSC has been obtained by reprogramming mature somatic cells using transcription factors or small-molecule compounds. Therefore, obtaining iPSC requires additional manipulation and is more cumbersome compared with other cell sources. PSC can be maintained and expanded as an undifferentiated clonal population for multiple generations. Undifferentiated hPSCs are usually maintained on feeder cells or specific ECM substrates. Since individual PSCs do not survive in vitro, PSCs are often harvested as cell aggregates, which maintains cell-to-cell contact and thus produces populations of cells with higher viability. Physical scraping also compensates for the problem of making up for uneven cell aggregation. To hinder cell separation from the culture plate, the dissociating enzyme mixture should be selected based on whether the cells are secreting too much ECM and the level of cell sensitivity [60].

After cell isolation, cells are usually inoculated into a biologically derived matrix (e.g., Matrigel), natural ECM (e.g., collagen), or a synthetic hydrogel. The composition of Matrigel is similar to that of basement membranes and consists mainly of basement membrane glycans, growth factors, nested proteins, laminin, and type IV collagen [61]. Although Matrigel can support organoid cultures, its inherent heterogeneity and ill-defined composition make it virtually impossible to control and improve the integration cues required for organoid cultures. Therefore, other matrices with defined compositions have been explored as alternative matrices to Matrigel, such as synthetic hydrogels [62], fibronectin [63], or recombinant human collagen [61]. To address the variation between Matrigel batches, natural matrices can be produced by recombination of proteins or polysaccharides [64]. Meanwhile, synthetic hydrogels have emerged as powerful tools to independently manipulate various properties of the substrate to control organoid-directed development. As an emerging engineered culture method for organoids, hydrogels will be elaborated on later.

Organoids can be derived from circular colonies produced by single cells [46,65] or initially multicellular structures (e.g., micropatterned cells [66], cell aggregates [67], or intestinal crypts [68]). The second method is for the early establishment of cell niches involving the same or different types of cells. The simplest method to form cell aggregates is to use ultra-low attachment petri dishes coated with hydrophilic hydrogel to prevent cell attachment [69]. Centrifugation enhances intercellular contact and promotes aggregate formation [70]. Microporous arrays can regulate the compaction and size of cell aggregates [71]. Another well-established method of forming cell aggregates is the hanging droplet method, in which the aggregates form at the bottom of the droplet. Previous studies have aggregated mouse cholangiocytes by droplet micro-fluidization to form complex organoids with hepatic mesenchymal cells [72].

Organoid culture is based on our accumulated knowledge of developmental biology [18]. Scholars must integrate various cues (e.g., cytokines, mechanical forces, and pH) to regulate organoid growth and development. As soluble cues, in specific temporal and spatial locations, researchers present various growth factors to cultured cells. In contrast to the original self-organizing organoid model, with the emergence and development of the engineered organoid concept, scholars can integrate cues to better control organoid morphogenesis. Cue integration is a common strategy used in the field of tissue engineering to construct tissues [73]. Ideally, simple, reproducible, and robust methods should be used to present specific physical or chemical cues in a spatiotemporal, physiologically relevant manner. Several methods (e.g., microporous arrays, microfluidics, hydrogels, bioprinting, and bioreactors) can be applied to design at different levels (e.g., molecular, cellular, and individual) to allow organoids to develop in the direction the researcher desires and meet the needs of regenerative medicine, ultimately creating the potential for clinical translation. We will elaborate on these later.

### Biological characteristics

Organoids from various sources exhibit common features during their cultivation, including the ability to increase the highly functional cell population. Cells in these organoids recapitulate the function and structure of the original organs. Notably, compared with iPSCs alone, organoids are uniquely scalable and suitable for large-scale expansion. Indeed, organoid construction offers various advantages, including shorter modeling times, individualization, and the potential for gene editing [74]. Hence, organoids overcome some of the shortcomings of other biological models and provide a valuable experimental basis for studying key functions in vivo. Moreover, in culture, organoids can efficiently develop region-specific features and, over time, generate various cells found in natural structures [45,75–79]. For example, in the nervous system, the cellular composition of cortical organoids is similar to that of the cerebral cortex [e.g., neuroepithelial (NE) and radial glial (RG) cells] [80–82]. Additionally, cells in the organoids follow the trajectory of organ differentiation [77]. For instance, neuroglia arise after neurogenesis, while astrocyte maturation closely mimics that of their in vivo counterparts [83,84]. In addition, organoids can generate vascular structures similar to that of the host [85,86], offering the possibility of long-term survival of organoids following transplantation. Finally, unlike traditional 2D cultures, organoids form under complex spatiotemporal regulation reminiscent of human embryogenesis, making them more suitable for transplantation therapy [33] and reducing the potential for immune rejection. In summary, the advantages of organoids address numerous challenges in regenerative medicine.

While avoiding ethical and allogeneic cell-related concerns, iPSC-derived organoids are not without their drawbacks, including low reprogramming efficiency and tumorigenicity [87]. These limitations have prevented the application of iPSCs in organoid cultures. One of the primary concerns with human iPSC-based therapies involves ensuring safety and preventing teratoma formation due to PSC contamination. A combination of transgene-free reprogramming and enzymatic dissociation facilitates teratoma-free transplantation of iPSCs in mice [88]. Pretreatment with an anti-CD30 antibody–drug conjugate has also proven effective in eliminating the tumorigenic potential of organoids without affecting their function [89]. These studies provide preliminary solutions to the tumorigenicity associated with iPSC-derived organoids. Mun et al. [90] presented a promising method for generating functionally mature human liver organoids derived from PSCs. This may represent a strategy to address the low efficiency typically associated with reprogramming organoids derived from iPSCs.

ESCs exhibit the remarkable ability to differentiate into various multicellular lineages, including mesoderm and endoderm. Liu et al. [91] discussed the advantages and limitations of a 2D hPSC-cardiomyocyte (hPSC-CM) system and presented recent advances in 3D culture platforms of hPSC origin. However, the utilization of ESCs requires the destruction of embryos, resulting in contentious ethical and moral dilemmas, limiting their applicability in organoid culture [92]. Similarly, reproductive cloning and immune rejection have prevented further application of ESCs [53].

ASC-derived organoids offer several advantages, including the ability to be cultured in vitro for long periods, a stable genetic profile, and rapid establishment and amplification capabilities. Moreover, they can be obtained directly from regenerated human adult tissues through a simpler and shorter procedure compared to human embryos. However, limitations in their differentiation spectrum and the existence of organ tissue cells with stem cell properties (redifferentiation potential) that cannot be obtained from adult organs hinder further research.

Different organoid stem cell sources have their own sets of advantages and disadvantages. Hence, researchers must carefully select cells in accordance with their research objectives. However, advances in bioengineering instill confidence that the future holds the potential to surmount the many limitations associated with organoids from different sources, thus enabling institutionalized, programmed, and scaled organoid cultures.

## Strategies for Culturing Engineered Organoids: Foundation for Application

Cultivating mature organoids with natural organ functions and physiological properties represents a key challenge for their application in tissue repair and biofunctional reconstruction. This section describes recently developed bioengineering tools that facilitate the maturation of engineered organs and form the basis for applications in tissue repair and biofunction reconstruction.

### Strategies for applying microwell arrays

To fabricate 3D multicellular plates, photolithography or micropatterning techniques are used. These methods involve using arrays of microwells with recessed portions of micrometer diameter that enable cells to form aggregates with specific parameters. This approach provides precise control over the formation of organoids and offers several advantages, including scalability, low cost, and ease of handling [16,93,94]. Chen et al. [95] used microwell arrays to develop mature human brain organoids, resulting in cultured products with ideal sphericity, size, fold index (sulcus), canal lumen size and thickness, and neuronal layer thickness.

**Fig. 1.**
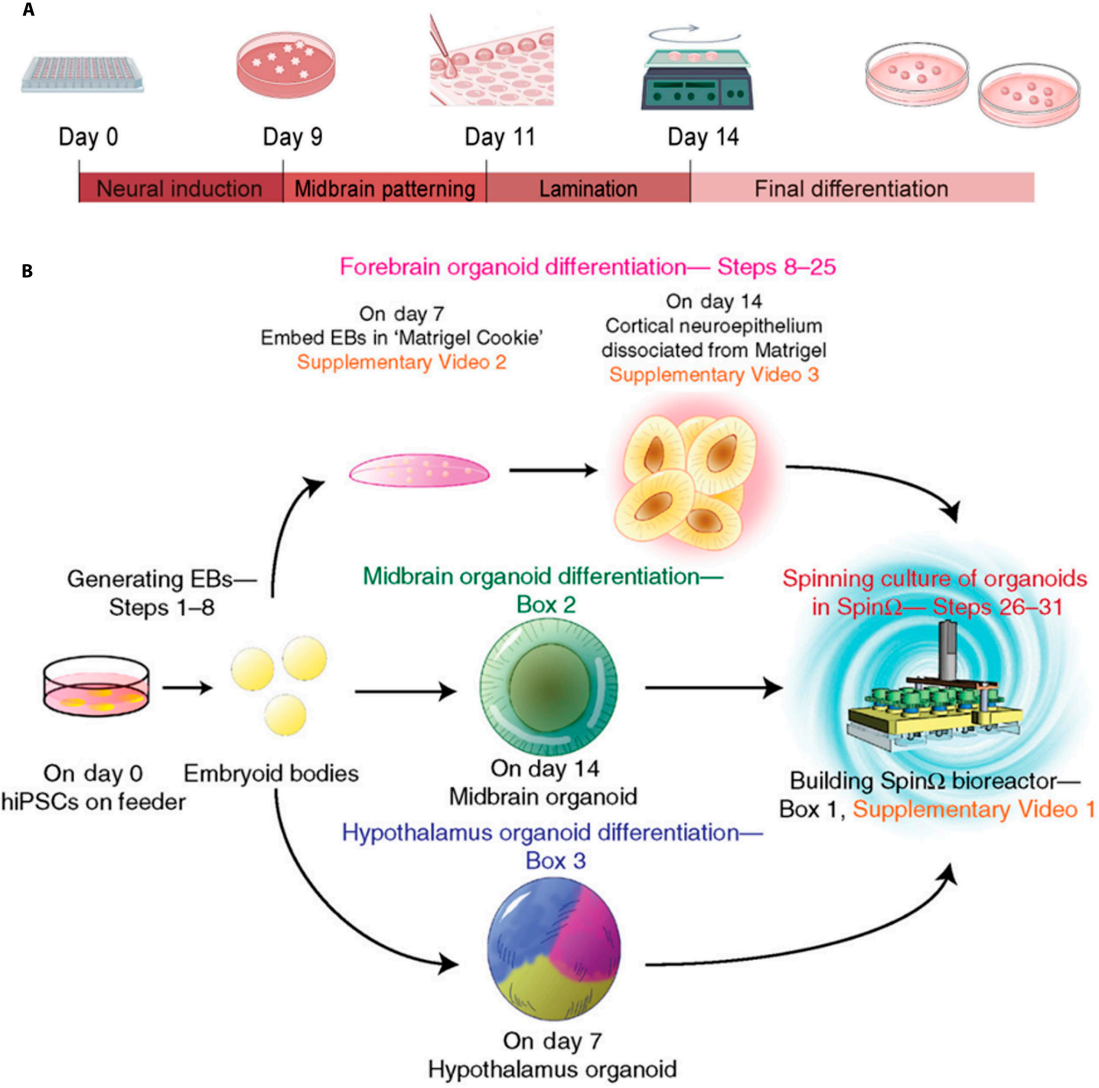
Strategies for culturing engineered organoids. (A) Schematic representation of the differentiation process from hiPSCs to hMOs by applying microwell array culture. Adapted reprinted with permission from [33], based on CC BY License, Copyright © 2023 Ivyspring International Publisher. (B) Schematic diagram of the scheme for the application of a customized bioreactor for the cultivation of specific brain organoids. Adapted reprinted with permission from [97], License Number: 5698250066668. Copyright © 2018, Springer Nature Limited.

Microwell arrays improve the consistency of organoid disease modeling. For instance, Zheng et al. [33] induced human midbrain-like organoids in low-cell-adhesion 96-well plates containing neurite-inducing medium; they were subsequently transferred to ultra-low-adhesion six-well plates containing the final differentiation medium (Fig. 1A). Meanwhile, Xue et al. [96] generated 3D-engineered human spinal cord organoids using PCSM-Matrigel@SAG. These porous chitosan microspheres (PCSMs) had uniformly distributed micropores. Cells developed within these microporous arrays, resulting in well-developed and homogeneous spinal cord organoids. However, using microwells for organoid culture requires tedious and repetitive experimental operations, reducing the culture’s efficiency. Artificial intelligence and robotics should be explored to enable efficient organoid culture.

### Strategies for applying bioreactor systems

The bioreactor system comprises a liquid-filled cylinder that rotates slowly on a shaft, moving the liquid and material in a circular path. Rotating bioreactors enhance material diffusion and can support the growth of nonvascularized 3D structures [97] (Fig. 1B). Cultivation of organoids using bioreactors is simpler than other methods while stabilizing the formation of well-morphologized organoids [98]. Przepiorski et al. [99] generated kidney organoids using a 125-ml rotating bioreactor, demonstrating that a low-cost method can produce organoids on a large scale while ensuring structural similarity with the original organs. Qian et al. [77] used a small rotating bioreactor system with a 12-well plate to generate forebrain-specific organoids. The resulting organoids have developmental characteristics of the human cerebral cortex, suggesting the potential of this culture system for efficiently generating organoids with intact structures.

Recently, Cohen et al. [100] developed a physical simulation method for hPSC culture using a stirred-tank bioreactor and achieved a 277-fold amplification rate in 6.5 days. This 3D culture system can potentially be used to improve the efficiency of the organoid culture. As culture technology advances, bioreactors are increasingly capable of facilitating large-scale, high-quality, efficient, and highly homogeneous organoid cultures.

### Strategies for applying microfluidic

**Fig. 2.**
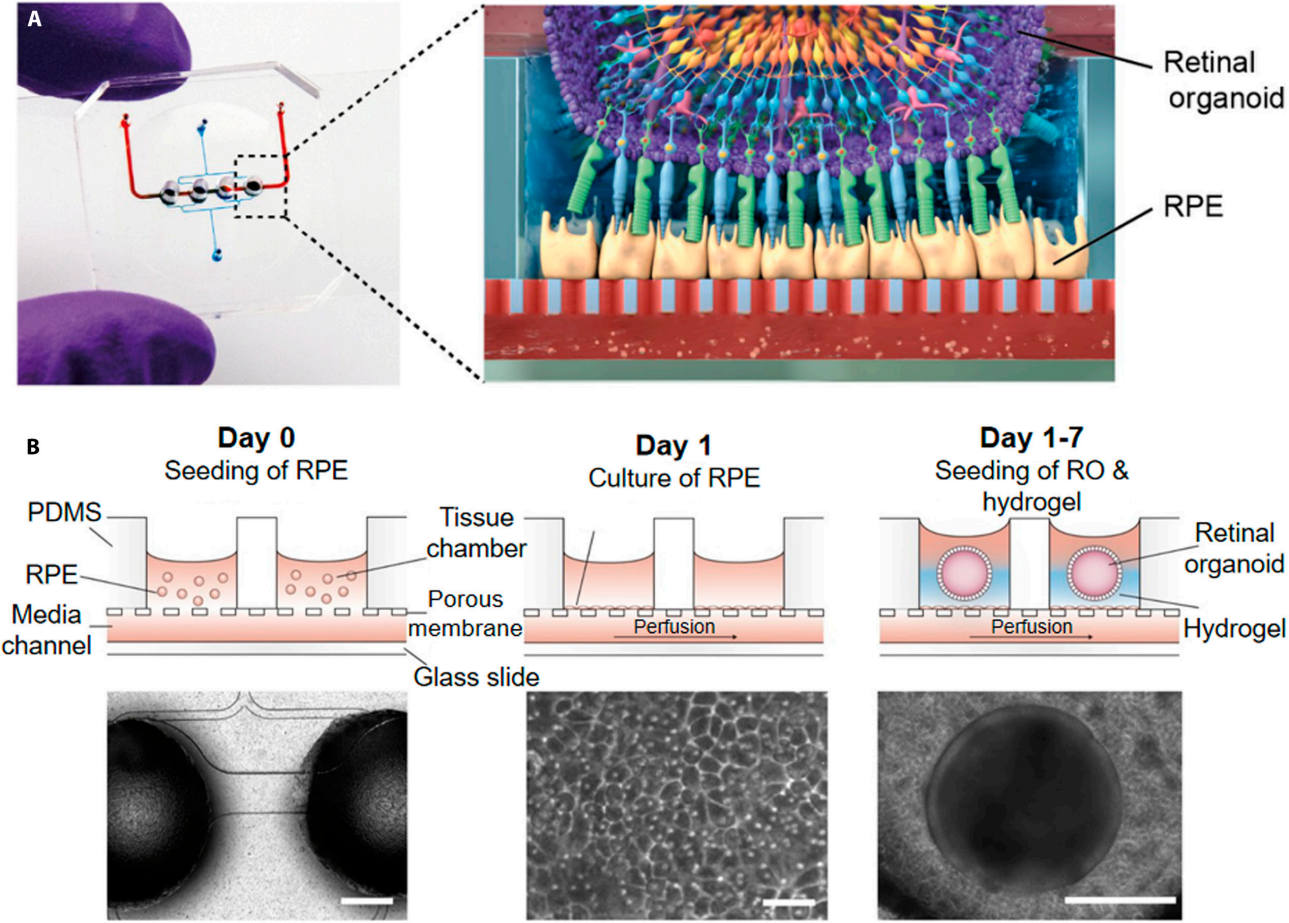
Application of microfluidic control to prepare the retina on a chip. (A) (left) Photograph of the retina-on-a-chip (RoC) and (right) schematic of retinal organoid photoreceptors interacting with retinal pigment epithelium. (B) (left to right) retinal pigment epithelium (RPE) cells seeded into the device (bars indicate 500 μm); dense monolayer formed after 24 h of incubation (bars indicate 80 μm); retinal organoid (RO) and hyaluronic acid-based hydrogel loaded directly into the wells from the top and then onto the RPE (bars indicate 400 μm) [103]. Adapted reprinted with permission from [103], based on CC BY License, Copyright © 2019, Achberger et al.

Microfluidics offers an effective system for forming and maturing organoids as they consistently provide the microenvironment, as well as chemical and mechanical cues needed by organoids [101]. Microfluidics enables higher-scale construction of organoids than traditional culture methods [102]. Combining microfluidic systems with “organoids-on-a-chip” facilitates the integration of multiple organs on a single platform [94]. Achberger et al. [103] designed organoid chips integrating more than seven basic retinal cell types (Fig. 2). Meanwhile, Tao et al. [104] developed multilayer microfluidic devices with through-hole polydimethylsiloxane (PDMS) layers and porous polycarbonate membranes to generate islet organoids. Indeed, the islet chip system is a powerful tool that provides real-time imaging and in situ tracking of islet organoid growth.

Integrating biomimetic materials into microfluidic devices has broadened the biomedical application range beyond the scope of what is possible with traditional microfluidic chips. For instance, Alamán-Díez et al. [105] constructed a collagen hydrogel-based bone-on-a-chip model using adipose tissue-derived stem cells. This represented the first miniature 3D in vitro bone model created from predifferentiated adipose tissue-derived stem cells embedded in a hydrogel collagen matrix for personalized bone tissue engineering. Sophisticated microfluidics and hydrogels have laid the foundation for in vitro models and the application of organoids in tissue repair. Microfluidics reduce cumbersome operations and increase the efficiency of individual organoid cultures. However, using microfluidics to achieve large-scale organoid cultures is challenging; hence, Abbasalizadeh et al. [106] used a scalable microfluidic platform for the serial production of highly functional vascularized hepatobiliary organoids from hPSCs. In addition, the multidimensional bioprinting technology facilitates more complex large-scale modeling. Meanwhile, machine learning, representing artificial intelligence, can efficiently analyze organoid cultures. Advanced imaging techniques (e.g., super-resolution microscopy) provide insights into cell development at the nanoscale. The seamless integration of these emerging technologies with microfluidic systems holds great promise for engineering organoid cultures. Due to the extensive research on organ-on-a-chip (OOC) systems, the ensuing intelligent OOC (iOOC) systems have recently gained increased attention. iOOC systems facilitate the creation of more controllable, dynamic, and accurate in vitro models, expanding our understanding of the structure and function of constructs.

### Strategies for applying bioprinting technology

**Fig. 3.**
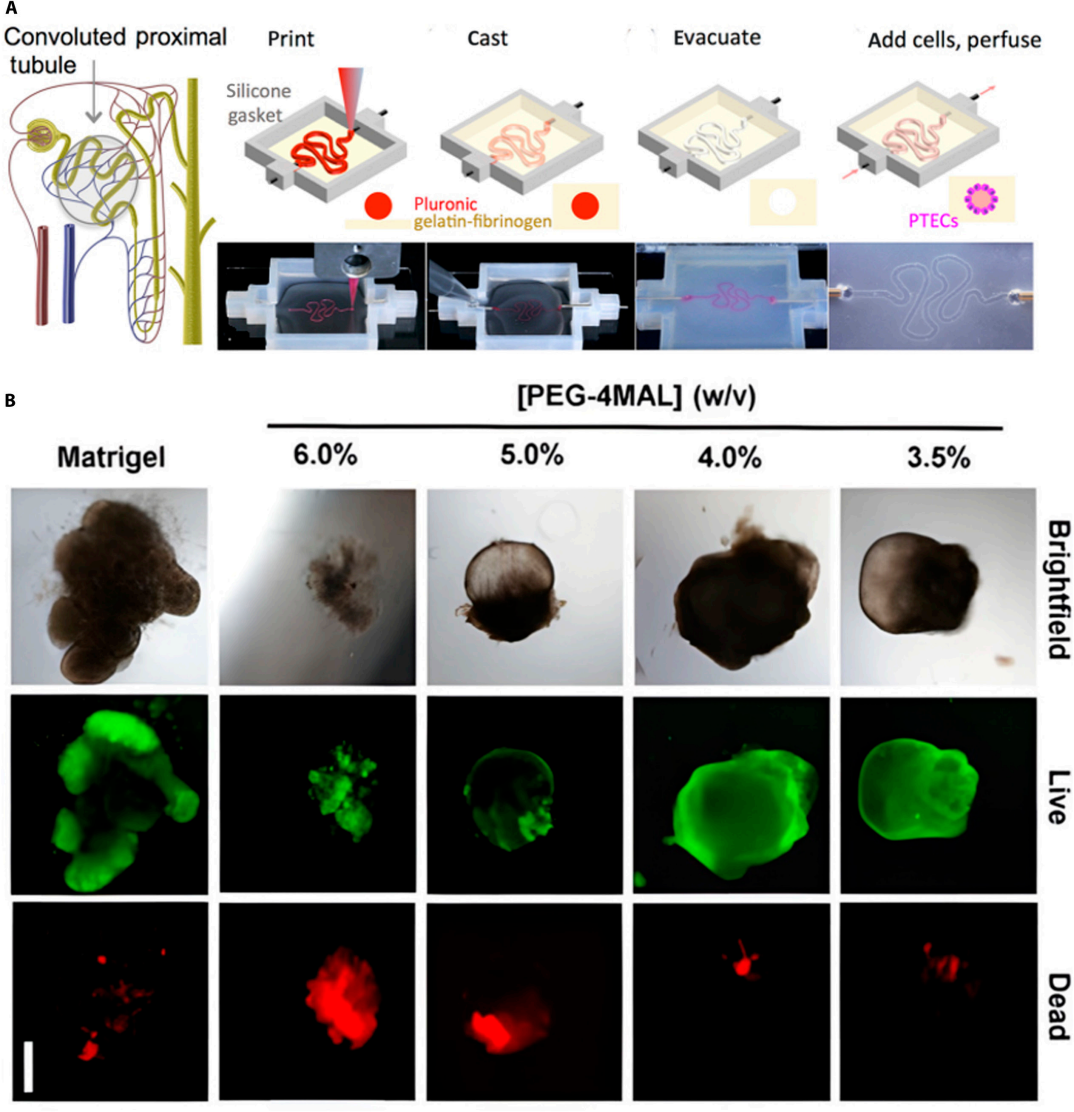
Strategies for culturing engineered organoids. (A) Corresponding schematics of the different steps in the fabrication of a 3D gyratory perfusable proximal tubule using 3D printing [109]. Adapted reprinted with permission from [109], based on CC BY License, Copyright © 2016, The Author(s). (B) Transmitted light and fluorescence microscopy images of HIO cultured in PEG-4MAL hydrogels or Matrix Gel at different polymer densities [62]. Adapted reprinted with permission from [62], License Number: 5698250436880. Copyright © 2017, Springer Nature Limited.

At the core of 3D bioprinting is bioink, a cell-containing hydrogel composed of ECM molecules and components that polymerize when exposed to specific stimuli, forming stable 3D structures after printing [107]. This technology can create 3D models with complex microstructures that are accurate and reproducible and hold great promise for culturing organoids [108]. Using bioprinting, Homan et al. [109] constructed functional kidney structures containing cell types exclusive to the kidney (Fig. 3A). Hence, bioprinting can be applied to create organoids with similar structure and function to the original organ. The bioink used by Kaur et al. [110] comprised a mixture of hepatocyte suspension and sodium alginate solution and was printed in a petri dish. These 3D bioprinted liver organoids formed clumps with liver-like functions. This method allows for the assembly of liver tissue layer by layer to form liver organoids while also facilitating the customization of the organoid shape, improving experimental precision and reproducibility. However, although bioink can be preserved relatively long, achieving vascularization and maintaining cell viability remain challenging. Therefore, combining bioprinting technology with hydrogel and microfluidic control may achieve complementary effects in organoid cultures.

4D printing allows biomaterials to alter their shape over time in response to stimuli (e.g., temperature, magnetic fields, pressure, electrical currents, and pH), better mimicking human physiological dynamics within tissues and organs [111]. Meanwhile, 5D technology adds another dimension by printing biological structures with higher mechanical strength, which holds promise for regenerating orthopedic and dental implants. Furthermore, 6D printing technology integrates the advantages of past technologies and is characterized by reduced resource consumption and shorter production times. Although it has not been extensively studied in the field of organoid culture, its potential provides exciting prospects for future research [112].

### Strategies for applying hydrogel matrices

When culturing organoids, hydrogels can be used alone or in combination with various culture techniques. Developing hydrogel matrices with stem cell biology offers opportunities to create more realistic organoids [113]. Hydrogel matrices are highly biocompatible with many properties that can be dynamically altered to closely mimic the physiological ECM [94,114,115]. Organoids cultured in specific hydrogels are superior to those lacking important components of the in vivo microenvironment (e.g., ECM). Indeed, growing awareness of the ECM has driven the use of customized hydrogels in organoid culture [116] due to their unique 3D structure and editable properties [117]. For example, Li et al. [118] used temperature-responsive poly(*N*-isopropylacrylamide) (pNIPAAm) hydrogels to fabricate spherical cell structures and generate bone organoids of bone marrow-derived mesenchymal stem cells (BMSCs) and BMSCs/dental pulp stem cells (DPSCs). In addition, Cruz-Acuña et al. [62] utilized hESCs and iPSCs to generate intestinal organoids, which were cultured in four-armed maleimide-capped polyethylene glycol (PEG) hydrogels and implanted in intestinal wounds via colonoscopy, improving colonic wound repair (Fig. 3B). PEG-4MAL hydrogels are powerful alternatives to Matrigel. This hydrogel-based model for specific organoid culture can be extended to other organoid applications in regenerative medicine. Interestingly, Treacy et al. [119] matured hiPSC-derived kidney organoids using fully synthetic peptide hydrogels with defined stiffness. Hence, a controlled growth environment is important for customized culture and maturation of organoids. Moreover, microenvironments with minimal complexity have utility for selective differentiation of kidney organoids. Rizwan et al. [120] developed customized viscoelastic hyaluronic acid hydrogels for culturing cholangiocytes and found that stress relaxation rates similar to those of liver tissue induced organoid growth of cholangiocytes and increased gene expression of Yes-associated protein. Combining certain microenvironment features with hydrogels for improved functionality creates the possibility of customized organoids.

In this way, it might be possible to customize “off-the-shelf” hydrogels for the direct culturing of specific organoids based on the patient’s needs without requiring the addition of induction factors or the use of cumbersome procedures [121].

## Exploring Modes of Organoid Engineering: Prospects for Development

Despite their success in culture, challenges remain regarding the application of organoids for tissue repair and biofunction reconstruction. However, the conventional organoid culture approach has not proven highly effective in achieving specific goals, such as directed differentiation, efficient amplification, and vascularization. Fortunately, these difficulties can be resolved using different levels of organoid engineering technology [122].

### Engineering organoids at the cellular level

**Fig. 4.**
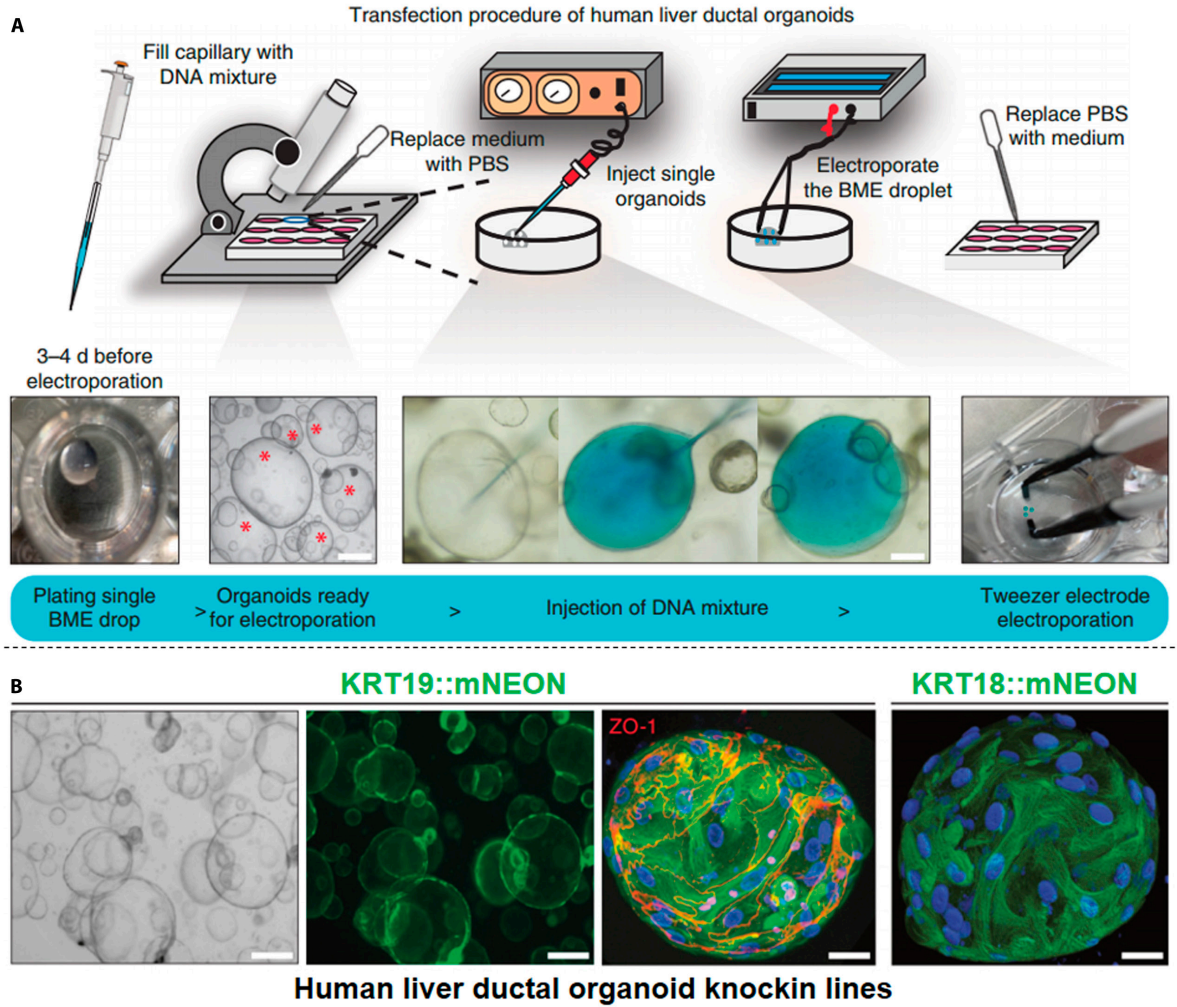
Genome engineering of human liver ductal organoids. (A) Schematic representation of the transfection procedure used. (B) Representative images of genome-engineered human liver ductal organoids. From left to right: Bright-field and fluorescent images showing a clonal KRT19::mNEON organoid culture, a KRT19::mNEON organoid stained for ZO-1, and a high-resolution image of a KRT18::mNEON organoid (bright-field image, 200 μm, and fluorescent images, from left to right: 200, 20, and 20 μm) [125]. Adapted reprinted with permission from [125], License Number: 5701080757553. Copyright © 2020, The Author(s), under exclusive licence to Springer Nature Limited.

Cells are essential elements of organoids and are ideal targets for manipulation. Modulating the properties of cells is a powerful strategy to improve organoid stability and enable customized applications. The desired endpoints can be customized by modifying the cell surface to improve or initiate cell aggregation in stable organoid cultures. Alternatively, gene editing techniques can be employed to control the intrinsic properties of cells by regulating key signaling pathway components or stem cell differentiation. For instance, Hogrebe et al. [123] found that regulating cells at an early stage of differentiation increases the efficiency of islet organoid generation. Targeting the cytoskeletal structure stimulates the targeted differentiation of iPSC into islet organoids. Meanwhile, Romitti et al. [124] forward-programmed the transient overexpression of transcription factors NKX2-1 and PAX8, triggering efficient thyroid differentiation and formation of functional follicles. Moreover, Hendriks et al. [125] employed CRISPR-Cas9 to generate adult hepatic ductal organoid systems. In turn, CRISPR-Cas9 and homology-independent organoid transgenesis (CRISPR-HOT) methods can also achieve effective gene knock-in (Fig. 4). Additionally, Cakir et al. [126] drove endothelial gene expression in stem cells to induce the vascular system, suggesting new strategies to overcome the organoid vascularization challenge. Intriguingly, Xu et al. [127] directly reprogrammed human astrocytes to generate a neural organoid for spinal cord regeneration. Genetic circuits can also be associated with cell fate selection. For example, Morsut et al. [128] designed the engineered system “synNotch” to enable experimental control of normal Notch signaling in cells by modifying the extracellular and intracellular structural domains of the Notch receptor, triggering directed cell differentiation. Meanwhile, Toda et al. [129] incorporated the synNotch system into a calmodulin-based intercellular adhesion control circuit involving two cell populations. The authors illustrated the possibility of integrating artificial gene networks in a multicellular environment and demonstrated the ability to trigger basic morphogenesis through a fully synthetic strategy.

Engineering organoids at the cellular level is emerging as a versatile strategy for precisely controlling cell fate and potentially customizing morphogenesis.

Recently, Wahle et al. [130] generated chromatin accessibility time-series datasets and single-cell transcriptomes, inferred the gene regulatory networks involved in organoid development, and investigated the overall patterns that regulate organoid development. Several studies have generated spheroids by manipulating cell collections and introducing them into organisms for further cultivation into engineered organoids. However, the current method for constructing engineered organoids using spheroplasts has certain limitations, such as matrix design and heterogeneity [131]. In addition, combining advanced technologies, such as optogenetics, can improve the customizability of engineered organoids by enabling precise spatiotemporal control.

### Engineering organoids at the niche level

Mimicry of specific niches is key to achieving stem cell self-organization in organoid culture. To expand cultured organoids and maintain their stability, bioengineering approaches can be applied.

#### Matrix design

The matrix provides support structures and nutrients for cultured organoids. Although contemporary developments of hydrogel matrices (e.g., Matrigel) have facilitated organoid development, the matrix origin is highly dynamic with an indeterminate composition and considerable heterogeneity. Hence, appropriate organoid matrices should exhibit high biocompatibility, excellent mechanical properties, and the ability to support cellular activity and mimic the specific microenvironment.

To date, myriad materials have been used to generate matrices (e.g., chitosan, collagen, hyaluronic acid, and gelatin); they failed to create the necessary ECM constituents.

Decellularized ECM (dECM) represents a prominent research avenue in a matrix design. Factors to be considered for the preparation of dECM include species origin, tissue origin, and the decellularization protocol. Several methods have been developed to obtain dECM, which has been used for tissue regeneration [132–135]. However, dECM must be combined with other components to form a hydrogel and ultimately a matrix for culturing organoids. Such hydrogels are multifunctional (e.g., heat-, light-, or pH-sensitive) and can enhance signals to guide cell behavior. The requirement for specific matrix components for different organoids requires on-demand customization, which alterable matrix designs can achieve. The development of bioscaffolds has led to a new era of tissue engineering, with dECMs receiving increased attention. Zeleniak et al. [136] used decellularized scaffolds to construct human thymic organoids, demonstrating the potential of thymic organoids to restore thymic function in patients. A protocol for bioengineering human intestinal grafts was designed by Meran et al. [137] Decellularized human intestinal scaffolds were generated from natural tissues and provided a cell regeneration system to form transplantable constructs.

Many aspects of dECM require further investigation, and the various components in dECM must be characterized and their biological roles must be established. Moreover, customized substrates must be developed and standardized to enable culturing of different organoids. Given that dECMs change in response to altered environments, determining whether they can be used as a basis for clinical research is necessary. Despite the numerous challenges, dECM remains a potentially ideal substrate for organoid construction [138].

#### Cellular interactions

Intercellular interactions also significantly influence the successful culture and directed differentiation of organoids. Romitti et al. [124] achieved efficient thyroid cell generation and avoided purification by maintaining the diversity of cells throughout the entire thyroid organoid culturing process. This emphasizes the critical role of nonthyroid cells in coordinating the full maturation of thyroid organoids in vitro and their seamless integration in the host body. Lei et al. [139] showed that alternating physical and molecular events between cells contribute to organoid morphogenesis, while environmental reprogramming allows cells to self-organize. Phase transitions at the tissue level can drive self-organization in organoid morphogenesis. Wu et al. [52] showed that CCL2 produced by immune cells can influence the immunoregulatory process of hair regeneration under mechanical stimulation and enhance cell allostery during organoid culture. This suggests that the interaction between immune cells in organoids and skin tissue cells affects hair growth. Meanwhile, Marchan et al. [140] found that MSCs promote the proliferation of functional chondrocytes in different dimensions of the culture system.

#### Controlling changes in space and time

The intrinsic capacity of stem cells drives organ formation. However, this capacity alone is not sufficient to produce fully functional organs. Sophisticated time- and space-varying stimuli are key factors influencing organ development in vivo. In conventional culture systems, the microenvironment in which cells grow is highly homogeneous, which does not correspond to a physiological growth state. This limitation can be resolved by releasing biomolecules in a spatiotemporally controlled manner [141–143]. Microfluidic devices can deliver morphogenetic substance gradients to artificially induce a controlled symmetry breaking [144–147]. Demers et al. [146] found that microdevices can be used to mimic in vivo gradients of morphogenetic substances [sonic hedgehog (SHH) agonists and bone morphogenetic proteins (BMPs)]. Substrates in the device can control ESCs to produce spatial differentiation similar to the in vivo development. Moreover, temporal control is required for the repetitive generation of desirable organoids. Utilizing photochemical principles facilitates the design of substrates that release target substances in response to light stimulation [148,149]. Light-activated release of localized morphogenetic substances may provide spatiotemporal control of crypt fossa-like bud formation in organoids. Additionally, to address changes in the mechanical properties of the ECM during intestinal organoid development, light-induced alterations in the mechanical properties of the substrate should be introduced. Similarly, scaffold shape could be altered over time by light-dependent in situ laser ablation to guide the development of custom-shaped organoids, as demonstrated by Pradhan et al. [150].

In conclusion, although engineering organoids at the niche level facilitates the maintenance of organoid culture systems, they generally exhibit heterogeneity and variability. Engineered substrates are expected to improve spatial and temporal control, mimicking the dynamic microenvironment of the original organ.

### Engineering organoids at the organism level

Organ development is influenced by the function of the surrounding tissues and system parameters (e.g., changes in oxygen levels, pH, and mechanical forces). Integration into the host environment is difficult for transplanted organoids; however, this adaptation is essential for the stable existence of the organoid [151,152]. Wang et al. [34] found that proper mesenchymal–epithelial interactions (MEIs) are necessary for hair regeneration in skin organoids in vivo after transplantation. This study provides important insights regarding mechanochemical coupling during self-organization, with implications for all organoid types. Sorrentino et al. [153] demonstrated that organoid growth is highly sensitive to stiffness, a mechanism independent of actin contraction. In contrast, abnormal matrix stiffness leads to an impaired proliferative capacity. Dynamic measurements of organoid–organism level parameters have also made significant progress. Mason et al. [154] developed an unbiased passive optical coherent elastography method that enables real-time noninvasive quantitative analysis of organisms, representing a milestone in the online assessment of spatial mechanical properties of organoids. Recently, Abdel Fattah et al. [155] utilized embedded magnetic nanoparticles to drive localized regions within organoids. Internal and localized mechanical forces generated by magnetic fields regulate the morphological maturation of organoids in vivo.

**Fig. 5.**
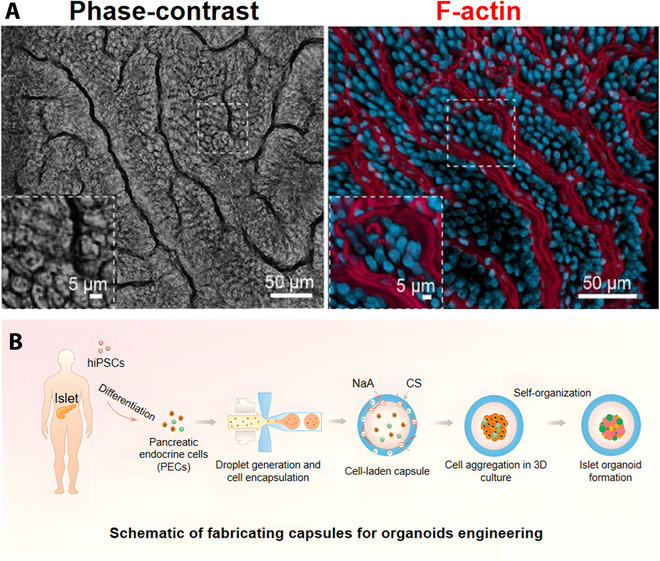
Exploring modes of organoid engineering: Prospects for development. (A) Representative IF microscopic views above intestinal epithelium grown on microarrays for 12 d under fluid flow and cyclic strain show the presence of continuous brush borders along the parietal membrane of epithelial villus-like protrusions that are juxtaposed in close proximity, as visualized by labeling the nuclei with F-actin (magenta) and labeling the cells with 4′,6-diamidino-2-phenylindole (DAPI) (blue) [162]. Adapted reprinted with permission from [162], based on CC BY License, Copyright © 2018, The Author(s). (B) Schematic of capsule fabrication for engineered organoids [172]. Adapted reprinted with permission from [172], based on CC BY License, Copyright © 2020 The Authors. Published by WILEY-VCH Verlag GmbH & Co. KGaA, Weinheim.

Several techniques for generating force and maintaining fluid flow can be realized on microfluidic platforms. However, their relevance has been questioned due to their inability to represent cellular diversity [156]. Nonetheless, the microfluidic platform enabled the cultivation of gastric organoids, allowing micropipettes to induce peristaltic-like motion through intraluminal flow and pressure cycling [157]. This system illustrates the potential for integrating biophysical factors into organoids using engineering methods, although the specific effects of these environmental stimuli have not been elucidated. Similarly, Homan et al. [158] utilized fluid flow on a chip to subject kidney organoids to shear stress. Fluid flow further improved the maturation of the physiological structure of kidney organoids and assisted in the generation of a functional vascular network. This device allowed epithelial cell growth on a 2D membrane with microfluidic channels that provided applicable fluid flow and bidirectional pathways [159–161]. Kasendra et al. [162] found that cells inoculated on microchips can grow into functional epithelia with villous folds when exposed to mechanical stretch cycles and luminal flow that mimic intestinal peristalsis (Fig. 5A). This micromachining-based device can combine physical and chemical stimulation, allowing for more stable organoid cultures with physiologically relevant properties.

In addition, most pathological changes and physiological mechanisms should not be attributed to specific organs as they result from the interactions of many organs or systems. OOC technology provides an important device basis to facilitate the integration of organoid technology with multi-organ engineering. Imura et al. [163] used a micromachining system to simulate the pharmacokinetics of anticancer drugs in vivo by combining intestinal and liver models with a breast cancer model. Meanwhile, Miller and Shuler [164] developed an engineered complex comprising 13 organs, referred to as a “body-on-a-chip.” Although the authors employed only five cell lines for proof of concept, the results showcased excellent tissue viability and basic physiological functions. Microfluidic cultures containing gastric, hepatic, and intestinal organoids recapitulate the interactions between organs to achieve paracrine signaling-dependent modulation of bile acid secretion [165].

In conclusion, the approach to obtaining high-quality organoids should consider organ niches and organism-level metrics. Parameters, including biophysical factors, biochemical signaling with other organoids, and transport of metabolic substances, that mimic the physiological state are expected to produce organoids that are mature in development, last longer, and have higher cellular diversity.

### Upgrading the characterization method

To establish homogeneity between constructed organoids and original organs, various characterization methods have been employed for different organoids. This involves assessing the morphology, structure, and function to provide evidence supporting the potential use of transplanted organoids in organ regeneration. The characterization of organoids serves as a vital tool for researchers, enabling them to ascertain whether cultured organoids meet their intended criteria. This not only paves the way for subsequent transplantation but also reduces the incidence of experimental setbacks and conserves research resources. Whether characterizing organoids before transplantation or evaluating posttransplant organoid function, researchers are actively exploring more advanced, noninvasive, and dynamic methods. The current limitations in maximizing the benefits of organoid systems stem from the absence of suitable characterization methods. Organoid research has relied primarily on characterizing their phenotypes. However, a comprehensive analysis of organoids requires versatile, accurate, and dynamic characterization methods that can be automated to run at high throughput.

Organoids are cultured in 3D matrices where their growth position is unregulated. Optical observation does not readily depict organoid growth via real-time dynamic imaging. To facilitate live cell imaging, microfluidic platforms or microwell capture cells can be used to engineer organoid growth sites [70,71]. Jin et al. [166] found that intestinal organoids can be captured in microcolumn arrays after aggregation to monitor morphological changes. To measure changes in physiological metrics during organoid culture, new imaging characterization methods have been developed. Okkelman et al. [167] found that continuous phosphorescence lifetime imaging microscopy using an O_2_-sensitive probe can track and reveal oxygen levels specific to intestinal organoids. In addition, Muta et al. [168] investigated the activity of intracellular proteins, particularly extracellular signal-regulated kinases, using engineering strategies based on the Förster resonance energy transfer.

Combining micromachining strategies and imaging methods allows ample organoids to be simultaneously analyzed [70,169], which is important for adequate pairing of patients and organoids. Microfluidic platforms allow preselection of organoids by size, reducing organoid variability and resulting in a more uniform mass. Droplet microfluidics is a promising cell encapsulation technology not yet integrated with organoid engineering technologies. Nevertheless, hydrogel capsules have great potential for customized organoid culture. For example, prostate and mammary cells embedded in matrix gel beads can be spatially and temporally controlled during differentiation and proliferation [170]. The application of a high-throughput, automated approach to generating cell-containing matrix gel beads [171] provided many microcapsules of uniform size, cell distribution, and composition, offering the potential to enable high-throughput characterization. Moreover, easily handled microbeads allow for the manipulation of individual organoids in a controlled manner. Liu et al. [172] encapsulated human iPSCs in hydrogel capsules, forming functional organoids with homogeneity (Fig. 5B). Using these methods, researchers can automatically generate large numbers of homogeneous organoids, setting the stage for automated large-scale analysis.

## Engineering Organoids with Applications in Tissue Repair and Biofunction Reconstruction: Possibilities for Clinical Translation

The need to address tissue damage due to various causes has spurred research into the development of functional substitutes for organs [173]. In regenerative medicine, advanced bioengineering techniques are fueling a growing interest in leveraging the potential of organoids developed [174]. Organoid transplants have demonstrated a higher degree of success in seamlessly integrating with patient tissues compared with stem cell-based approaches [175,176]. Organoids encompass stem cells with the potential to mature into fully developed organs, offering a promising source for organ transplantation. The attainment of mature organoids is characterized by morphology, structure, and functionality that are nearly indistinguishable from normal organs. In this section, we explore organoids applied to regenerative medicine, discuss their preparation, characterization methods, and significance, and demonstrate the design ideas of different organoids. Organoids were successfully transplanted into animal models or humans and performed certain physiological functions (Table). In addition, although other engineered organoids have not yet been applied in regenerative medicine (i.e., have not been transplanted into the organism or have not performed normal physiological functions after transplantation), they have a significant potential for clinical translation.

**Table.** Organoids with potential for regenerative medicine

Target organ	Stem cell type	Results of studies	Reference
Brain	iPSC	Transplanted hMOs improve motor function in PD mice.	[33]
Brain	ESC	Transplanted brain organoids not only form specific cortical structures but also achieve improved neuromotor function in stroke mice.	[177]
Brain	iPSC	Transplanted organoids form synaptic connections with the host visual system and retina. Flash visual stimulation of rats also elicited responses from neurons in the organoid.	[178]
Spine	iPSC	Organoids differentiate into neurons and form synapses for normal connectivity at the site of lesions in mice with complete spinal cord injury.	[127]
Skin	ASC	Sweat gland organoids promote sweat gland regeneration and wound healing in the body.	[179]
Skin	iPSC	The organoids was successfully transplanted into a mouse skin injury model and developed into fully functional sweat gland organoids in vivo.	[180]
Mesenchymal	iPSC	Transplanted organoids enhance epidermal stem cell activity in the skin of mice with limited scleroderma and promote regeneration of blood vessels and sweat glands.	[181]
Skin	PSC	The cultured “human skin” grew a notable amount of “hair” on previously hairless nude mice.	[182]
Bone	iPSC	Organoids mediate successful bridging of middle and long bone defects in immunocompromised mice.	[183]
Cartilage	ASC	Osteo-callus organoids enable rapid bone regeneration in as little as 4 weeks in rabbits with large bone defects.	[184]
Cartilage	iPSC	In a primate model of cartilage defects in the knee joint, allogeneic iPSC-derived cartilage organoids did not elicit an immune response in cartilage defects and directly promoted tissue repair for at least 4 months.	[185]
Bile duct	ASC	ECOs rebuilt the gallbladder wall and repaired the biliary epithelium, settled in their physiological ecological niche, and performed their original physiological functions without any complications.	[186]
Liver	iPSC	hiPSC liver organoids attach to and repopulate rat liver parenchyma and secrete human albumin.	[187]
Bile duct	ASC	Provides proof of principle that cholangiocyte organoids can be used to repair human bile duct epithelium.	[23]
Pancreatic islet	ASC	It was able to reverse the disease when transplanted into diabetic mice.	[188]
Thyroid	ASC	Thyroid-like follicles produced after transplantation into the subperitoneum of the kidney of hypothyroid mice are functional structures capable of secreting thyroid hormones.	[189]
Thymus	iPSC	Humanized mice receiving thymus organoid transplants can mediate cellular and humoral immune responses.	[136]
Pituitary	PSC	Human PSC-derived pituitary organoids show hormone release in vivo after transplantation into a mouse model of hypopituitarism.	[190]
Intestine	ASC	Intestinal organoids can be successfully transplanted in situ, and mice with successful transplants regained their body weights significantly.	[191]
Intestine	ASC and ESC	Transplantation of intestinal organoids into the colon of recipient mice forms the basis of the first human clinical trial utilizing colonic organoid transplantation therapy for the treatment of refractory ulcerative colitis cases.	[192]

### Brain organoids

Due to its subtle nature and extraordinary cognitive abilities, the human brain is often considered the most complex biological structure. Hence, any damage inflicted upon it can result in profound alterations in neurological and psychological health. Meanwhile, the gap between humans and animals impedes the understanding of the complexity of the human brain. Although studies of model organisms, such as mice, fish, and other vertebrates and invertebrates, have provided insights into the structure and function of the brain, the study of human neurobiology and related diseases remains a formidable challenge. Currently, engineered brain organoids provide the material basis for the treatment of refractory brain diseases, with the hope of repairing and functionally reconstructing the brain. Parkinson’s disease (PD) is a refractory neurodegenerative disease afflicting an increasingly younger population. Zheng et al. [33] transplanted human midbrain organoids (hMOs) at different stages of differentiation as tissue blocks into the striatum of naïve immunodeficient PD mice. Assessment of axonal innervation and cellular activity in vivo showed that transplantation of human iPSC-derived hMOs improved motor function in PD mice. This highlights the potential of hMOs as effective grafts for treating PD. Regarding brain organoids, ensuring that the developmental status of brain tissues and the expression of characteristic cellular markers are normal during culture is necessary. Zheng et al. [33] analyzed hMOs using reverse transcription polymerase chain reaction (RT-PCR) and immunofluorescence (IF) staining. After 15 days, hMOs stained positively for various neuronal cell-specific markers (e.g., embryonic midbrain progenitor cell marker OTX2). Evidence suggests that early hMOs primarily comprise midbrain dopamine (DA) lineage cells, providing insights for future reconstruction of midbrain function in PD patients using organoids.

Characterizing the substances released by brain cells can help to determine whether organoids have certain physiological functions. Wang et al. [177] transplanted hESC-induced brain organoids into a rat model of middle cerebral artery occlusion that not only formed specific cerebral cortical structures but also improved neuromotor function in stroke mice. This suggests the great potential of brain organoids for tissue repair and biofunctional reconstruction. Meanwhile, Xue et al. [96] utilized calcium delay imaging to examine calcium signaling dynamics. Treatment with high potassium chloride induced a burst of calcium activity, potentially modulated by 6-nitro-sulfamoyl-benzo-quinoxaline-dione (NBQX), demonstrating specific neural network activity in these organoids. Cao et al. [178] used cylinder, grid walking, and deglutition tests to assess the recovery of sensory and motor functions in stroke mice transplanted with human brain organoids and showed that neuronal activity of transplanted organoids was critical for the recovery of sensory-motor functions after stroke.

Vascularization of brain organoids is a challenge that must be addressed to achieve long-term survival after transplantation. Current vascularization methods usually produce a network of endothelial cells surrounding organoids. However, the locations most needing perfusion are centers with no vascular distribution. These vasculature-like structures contain luminal space; however, they lack the transport mechanisms of real blood flow. Furthermore, in attempts at vascularization, many scholars have emphasized the culture of endothelial cells without incorporating the influence of other cells (e.g., astrocytes involved in blood–brain barrier formation). Wang et al. [179] transplanted pericyte-like cells into organoids to observe the interaction between astrocytes and pericytes, which form basement membranes and neurovascular-like units.

Xu et al. [127] reprogrammed human astrocytes and formed spinal cord organoids by adding inducible factors. In mice with complete spinal cord injury, the organoids transplanted to the lesion site differentiated into spinal cord neurons and migrated and formed synapses with host neurons, realizing normal connections between the organoids and host structures. Following the transplantation of a human brain organoid into a large, injured cavity in the visual cortex, Jgamadze et al. [180] demonstrated its ability to integrate with the adult rat visual system. Visual stimuli to the host animal elicited responses (e.g., orientation selectivity) from organoid neurons. Virus-based transsynaptic tracings revealed multi-synaptic pathways between organoids and the host retina, as well as interconnections with other regions of the visual system.

### Skin organoids

Skin is the largest human organ, of which the mechanisms of repair following injury are complex [181]. Injuries extending into the dermis can result in poor healing and unsightly scars due to a lack of keratinocytes [182]. Engineering organoids offer a promising modality for skin repair. Lee et al. [183] generated neural tissue using co-development of dermal and epidermal cells. Although the results did not produce many accessory structures, such studies are rare in the field of skin organoids, warranting further elucidation of the underlying mechanisms. Lei et al. [139] characterized basal epidermal cells by immunostaining for K14 and K10 and performing hematoxylin and eosin (H&E) staining. Results showed morphological changes in these skin structures during culture, demonstrating six successive stages of self-organization of skin-like organoids. Meanwhile, Lee et al. [183] stained skin organoids on day 24 of culture, confirming that the skin organoids were in a normal developmental cycle based on cell surface markers.

Skin appendages influence various aspects of mammalian life. With more than 30% of the world’s population suffering from varying hair loss, establishing methods to achieve hair regrowth has become a booming research area. Assessing hair growth status after transplantation is important to assess the function of skin organoids. To this end, Wu et al. [52] transplanted skin organoids into the backs of nude mice and observed that the effect of hair regeneration was significantly improved compared with the control group. The authors also assessed how mechanical stimulation affects the activity of hair follicle stem cells using a plucking model. Cells from day 3 after plucking had the strongest hair regeneration capacity after transplantation and could self-organize during organoid culture. Meanwhile, Lee et al. [183] found that skin organoids spontaneously produce neonatal hair follicles as they grow, laying the foundation for constructing skin organoids for skin tissue repair.

Lee et al. [184] successfully cultured skin organoids with intact neural circuits and skin appendages using hPSCs over 140 days. When grafted onto nude mice, the cultured “human skin” grew a notable amount of “hair” on previously hairless nude mice. This groundbreaking achievement represents the world’s first complete “skin” cultivation.

Diao et al. [185] constructed sweat gland organoids, which, after transplantation into sweat gland-damaged mouse skin, effectively integrated into the tissue and participated in sweat gland regeneration and wound healing. Sun et al. [186] demonstrated a practical and feasible strategy to generate sweat gland organoids and treat or replace damaged skin tissues using reprogramming methods. Ma et al. [187] constructed mesenchymal organoids that can promote the recovery of skin-related functions and scleroderma pathological phenotypes, hinting at the potential of regenerative medicine for the treatment of scleroderma.

### Bone organoids

Human bone has a high capacity for regeneration; however, the sources of materials used in bone surgery remain limited [188,189]. The bone microenvironment (including multiple cells, ECM, soluble growth factors, and cytokines) is the foundation for bone organoids and facilitates bone marrow replication [190]. Auto-transplantation of amplified bone organoids provides an important resource. Dai et al. [191] developed a strategy for in vivo bone organoid generation triggered by BMP-2 utilizing biologically active materials.

This in vivo bone organoid develops bionically to form a bone marrow-like structure containing myriad therapeutic cells (e.g., mesenchymal stem cells, various immune cells, and hematopoietic stem cells). The bioactive components produced by these bone organoids can offer hope for reestablishing physiologic function in patients with bone marrow disorders.

The complexity of bone makes constructing bone organoids for tissue repair and biofunctional reconstruction challenging [192]. Nevertheless, Nilsson Hall et al. [193] reported an engineered healing tissue organoid capable of spontaneously bioassembling into large, engineered tissues that healed long bone defects in mice. The morphology of cultured regenerated bone is similar to that of natural tibia. Tam et al. [194] used CTAn (Bruker micro-CT, BE) for image processing to quantify the mineralized tissue and detect progressive mineralization. Masson’s trichrome staining revealed active bone growth, particularly at the cartilage–bone transition zone. Meanwhile, Tam et al. [194] prepared cartilage organoids and found that they could repair long bone defects without scaffolding and had an indirect effect on bone regeneration. Characterization methods can be employed to detect the assembly and maturation processes of bone organoids. Nilsson Hall et al. [193] used filamentous actin and 5-ethynyl-2’-deoxyuridine staining to observe the process of endochondral ossification. The authors analyzed the gene expression of related markers and found that the expression of the transcription factor osterix and early osteogenesis marker runt-related transcription factor 2 was up-regulated.

Significant strides have been made in the cultivation and transplantation of bone organoids, leading to successful long-bone repairs after transplantation [193,194]. However, the challenge of long-term in vivo survival remains to be addressed. Li et al. [118] successfully created vascularized scaffold-free bone organoids with potential applications in regenerative medicine using cell cultures containing only BMSCs.

The regenerative capacity of human cartilage is considerably weak compared with bone tissue; thus, access to raw materials for cartilage repair is necessary, particularly after reconstructive surgeries of related organs [140,195]. The application of cartilage organoids has been extensively investigated. For example, stem cell differentiation has been induced within scaffolds to construct cartilage organoids in vitro, which were then implanted into cartilage-injured mice to successfully repair damaged tissues [196]. Xie et al. [197] utilized a stepwise induction technique and digital light processing printing to synergistically load hydrogel microspheres with BMSC. After chondrogenic induction, the microspheres were aggregated into osteo-callus organoids, which showed higher chondrogenic efficiency than conventional microspheres. The osteo-callus organoids transplanted into rabbits also achieved rapid bulk bone regeneration. Abe et al. [198] demonstrated the integration of allogeneic iPSC-derived cartilage organoids and their remodeling into articular cartilage in a primate model of knee cartilage defects. iPSC-derived cartilage organoid xenotransplantation may have potential clinical applications in the treatment of articular cartilage defects.

### Liver and bile duct organoids

The liver can regenerate, even if it loses most of its parenchyma, and withstand long-term damage. However, hepatocyte proliferation and liver regeneration diminish with liver injury. Due to the lack of effective treatments for liver failure, new regenerative strategies are needed that stimulate and support endogenous repair mechanisms to rejuvenate or replace the original liver [37]. Engineered organoid technology has significant potential to replace the original damaged liver and restore normal physiology and function [199]. Organoid-based regenerative medicine therapies can also be used for the ex vivo repair of liver grafts to increase the supply of transplantable liver tissue [37].

In groundbreaking research in 2013, Huch et al. [65] established liver organoids that could expand and self-renew over time. Subsequently, Peng et al. [200] and Hu et al. [201] developed a system to utilize hepatocytes for long-term culture of organoids. Importantly, cultured organoids have enhanced implantation and regenerative capabilities, exhibiting promise for applications in regenerative medicine. However, most of the work has been performed in vivo in mice, and differences have been reported in the growth of cultured organoids from different tissue sources [200,201].

Sorrentino et al. [153] found that liver organoid growth is sensitive to stiffness and requires the activation of yes-associated protein 1 (YAP) and src family of kinases (SFKs), a mechanism that is independent of the actin contraction. Meanwhile, culturing liver organoids requires attention to the surface molecular expression of organoid cells.

Wu et al. [202] analyzed the expression of hepatocyte-specific markers (e.g., SOX9, HNF4α, and AAT) via IF and flow cytometry. The proportions of mature hepatocytes and cholangiocytes identified at late culture stages using flow cytometry were approximately 48% and 28%, respectively. Moreover, Tanimizu et al. [203] found that labeled bile acids are taken up by hepatocytes, excreted into the bile ducts, and accumulate in the biliary system. Using phase contrast microscopy, the authors identified tubular connections between cholangiocytes and hepatocytes. This constructed organoid was designated the “hepatobiliary tubular organoid (HBTO)”. Wang et al. [204] transplanted expandable liver organoids derived from hESCs into the epididymis of immunodeficient combined diabetic mice and analyzed them. H&E staining showed consistent formation of ductal epithelial structures.

Liver organoids will help to facilitate the cost-effective production of valuable biologics like albumin and clotting factors. Organoids transplanted into the body are capable of biofunctional reconstruction in patients with liver diseases. Indeed, cocultured hepatobiliary organoids have been developed, confirming the possibility of normal organ function after transplantation [202,203]. A significant breakthrough was achieved by Kaur et al. [110] with the 3D bioprinting of liver organoids, proposing a potential treatment for liver failure. Similarly, Wang et al. [205] aimed to bioengineer the creation of human liver lobular structures by cultured endothelial cells and hepatocytes on lobule-like scaffolds. The coculture of hepatocytes resulted in superior results to monolayer culture, with significantly enhanced functions (e.g., secretion of proteins, substance metabolism, and synthesis of urea).

Takeishi et al. [206] used human skin cell reprogrammed iPSCs to create fully functional miniature livers, which survived for 4 days when transplanted into rats. These miniature livers also secreted bile acids and urea, similar to normal liver; liver maturation was achieved in less than a month, a process that typically takes up to 2 years naturally. Hence, in the foreseeable future, conditions such as acute liver failure may necessitate only a period of intensive liver therapy rather than an entire liver transplant. Compared with primary cholangiocytes, extrahepatic cholangiocyte organoids (ECOs) cultured by Sampaziotis et al. [207] are very similar in terms of functional properties and transcriptomic features. Following transplantation into a mouse injury model, ECOs reconstructed the gallbladder wall and repaired the biliary epithelium. The authors demonstrated the integration of ECO-reconstructed gallbladder and bile ducts using IF, histology, and quantitative PCR analysis. The transplanted cells expressed biliary markers (e.g., CK7, CFTR, and SOX9). Histological and IF analyses revealed an open lumen and formation of biliary epithelium by the transplanted green fluorescent protein–positive (GFP^+^) cells. IF analyses also revealed the absence of GFP^+^ cells in the adjacent hepatic tissues, and that the scaffold was colonized by endogenous cells after transplantation. This suggests a high degree of integration of the organoid into the organism’s microenvironment without invasion of surrounding normal tissues. The integrity of the reconstructed gallbladder and patency of the regenerated lumen were demonstrated using magnetic resonance cholangiopancreatography (MRCP) imaging and confirmed using fluorescein Isothiocyanate (FITC) cholangiography. Whole-body magnetic resonance imaging and autopsy examination at 104 days after transplantation showed no evidence of tumor formation. In addition, 1 month after transplantation, the authors observed minimal apoptosis and proliferation in the transplanted ducts, confirming the stability and integrity of the reconstructed bile duct epithelium. Mice receiving ECOs showed no increase in serum cholestatic markers (e.g., bilirubin and alkaline phosphatase [ALP]), providing ample evidence that ECOs maintained normal metabolic function of the liver and gallbladder in vivo in injured mice. These results indicated that ECOs can colonize and regenerate portions of the biliary tree in their physiological ecological niche and perform original physiological functions without any complications (e.g., carcinoma). Tsuchida et al. [208] safely transplanted liver organoids through the portal vein, which can completely replace chronically damaged livers and reduce precancerous lesions. hiPSC liver organoids are superior to monolayer cells in bile duct reconstruction and liver regeneration and can fill the damaged liver parenchyma and secrete human albumin. Using a novel model of cell transplantation in human organs and a mouse model of biliary tract injury, Sampaziotis et al. [23] demonstrated that the plasticity of bile duct organoids allows cholangiocytes from one region to repair a different region of the biliary tree, paving the way for the use of the organoids for cell-based therapies.

### Endocrine organoids

The endocrine system is closely related to realizing normal physiological functions in the human body. Due to its complexity, understanding and treating endocrine diseases have been a challenge. However, the emergence of endocrine organoids has opened up the possibility of repairing tissue damage after transplantation [38].

In 2014, Pagliuca et al. [209] reported a protocol for differentiating glucose-responsive β cells from iPSCs. Similar to the behavior of primitive pancreatic islets, iPSCs can differentiate from PP2 cells to SC-β cells. In addition, researchers have explored genetic modifications and reduced tumorigenicity to safely and efficiently culture organoids. The organoids are mainly composed of β cells that secrete adequate amounts of insulin [210]. Romitti et al. [124] found that thyroid-stimulating hormone and 3D culture influence the formation of ESC-derived thyroid organoids. The authors successfully prepared functional human thyroid-like organoids, evaluated their function in hypothyroid mice, and used this method to generate patient iPSC-derived thyroid organoids. Ogundipe et al. [211] explored patient-derived thyroid organoids for thyroid regeneration. These thyroid organoids were transplanted into the kidney capsule of hypothyroid mice. The thyroid-like follicles produced after transplantation were thyroid-functioning structures capable of secreting thyroid hormones.

Characterizing functionally diverse endocrine organoids is essential, considering both cell-specific markers and the physiological functions of these organs. Pagliuca et al. [209] stained human pancreatic β cells in vitro after antigen repair. The homogeneity of cultured cells with human pancreatic islet β cells was determined using immunohistochemical techniques. The in vivo function of hESC-derived thyroid organoids was evaluated by Romitti et al. [124]. Macroscopic and histological assessments showed that the transplanted organoid in the kidney was successfully implanted in the host microenvironment. H&E staining revealed that many follicles were organized in thyroid-specific structures. Thyroid function in mice was also improved after transplantation. The presence of blood vessels and stromal cells near thyroid follicles makes proximity to blood vessels critical for the release and targeted transport of thyroid hormones. T4 immunostaining showed that the functional recovery of transplanted follicles is a continuous process. Finally, the authors assessed the systemic effects of organoid transplants, demonstrating that hESC-derived thyroid follicles function normally in vivo.

Zeleniak et al. [136] utilized decellularized scaffolds to construct human thymus organoids. Thymic epithelial cells in organoids support the development of T helper cell subsets and T cell selection after transplantation into humanized mice. T cell-mediated humoral and cellular immune were generated. Based on previous differentiation methods, Kasai et al. [212] introduced smoothened agonist (SAG) and BMP4, which induced human iPSCs to generate structures with hypothalamic and pituitary functions. The induced pituitary gland showed adrenocorticotropic hormone (ACTH) secretory capacity and responded to low-glucose stimulation, comparable to that of adult mice. As a departure from previously reported differentiation conditions, Zimmer et al. [213] used a relatively simple method to derive a human pituitary lineage from human iPSCs. The resulting pituitary cells were stressed and could release hormones. The authors also assessed hormone release in hypopituitary mice after the transplantation of organoids. Alves-Lopes et al. [214] developed a three-layer gradient system for generating rat testicular organoids. This system generates germ cells and forms a functional blood–testis barrier in rat testicular organoids. In addition, dECM has been used to culture testicular organoids. Testicular cell suspensions from porcine immature testicular tissue have been used to form testicular organoids. They produced structures similar to spermatogenic tubules in hydrogels [215]. Mall et al. [216] developed a new method to generate heterologous organoids by combining human iPSC-derived primitive germ cell-like cells with rat testicular cells.

In summary, progress has been made in transplantation therapy using endocrine organoids due to the importance of endocrine function. Endocrine organoids remain in their infancy, and much research has focused on the cultivation of islet organoids to treat diabetes mellitus. Although endocrine non-islet endocrine organoids showed promising results in preclinical studies on restoring endocrine function, clinical studies have not been conducted. Utilizing organoid engineering techniques is important to ensure sustainability and safety. Current clinical translation of endocrine organoids remains challenging (e.g., costly growth factors and tumorigenicity of iPSCs). Utilizing bioengineering strategies (e.g., using 3D scaffolds to induce stem cell differentiation or gene editing to selectively eliminate cancer-associated cells) may be beneficial in accelerating the clinical translation of endocrine organoids.

### Intestine organoids

The intestinal epithelium limits the entry of microorganisms and harmful substances into the bloodstream. Therefore, damage to the intestinal epithelium inhibits the absorption of nutrients and allows pathogens to infect the organism, leading to various clinical manifestations. For example, patients with severe short bowel syndrome (SBS) [217–219] or congenital disorders [220] experience malnutrition due to reduced or dysfunctional intestinal absorption. Although several methods have been explored to restore intestinal function [221,222], they have significant limitations. Recent advances in engineered organoids have opened new avenues in the field of regenerative medicine, offering potential solutions for severe intestinal diseases [223].

Watson et al. [224] have demonstrated that hPSC-induced human intestinal organoids (HIOs) in vitro can be transplanted into immunodeficient mice. The transplanted HIOs underwent an evolution from fetal-like to adult-type features, indicating the influence of the in vivo environment on organ maturation. The authors reported increased expression of specific marker proteins in differentiated cells. Finkbeiner et al. [225] extended this work by showing that transplanted HIOs developed into a structure resembling the adult intestine, which was then implanted into the peritoneal cavity of mice as tissue-engineered small intestines (TESIs).

Organoid technology could enhance the pool of stem cells in culture and complement the existing TESI system by facilitating surgical connections to the natural intestine. Utilizing hPSC-derived neural crest cells, Workman et al. [226] integrated the nervous system of the gut in HIOs, providing an attractive strategy for obtaining a functionally intact gut to benefit patients with severe SBS. More recently, Sugimoto et al. [227] investigated an organoid-based organ repurposing method for treating SBS.

Yui et al. [228] demonstrated for the first time that intestinal organoids could be successfully transplanted orthotopically when transplanting colonic epithelial organoids. The weight of mice with successful transplantation was significantly restored. Using the same transplantation protocol, endodermal progenitor cells [229] and mouse intestinal epithelial organoids [230] were transplanted into the colon. Fukuda et al. [231] found that the small intestinal epithelial organoids transplanted into the colon appeared to behave differently. The regenerative capacity of adult small intestinal organoids was not affected even in the colonic environment, and the small intestine-specific structure and function were maintained. Sugimoto et al. [232] developed an effective in situ xenotransplantation method for human colonic organoids, utilizing gene editing techniques that allow for the continuous implantation and reconstruction of human colonic epithelium in the same anatomical location. These findings provide insights into the application of organoid xenograft systems.

However, the effectiveness of this method in other parts of the gut remains to be confirmed. Watanabe et al. [233] transplanted intestinal organoids into the colon of mice, which became the basis for the first human clinical trial utilizing colonic organoid transplantation therapy to treat cases of refractory ulcerative colitis. Following this, the authors transplanted organoids cultured using the patient’s healthy intestinal mucosal stem cells into a patient with refractory ulcerative colitis. The organoids were transplanted into the diseased area of the large intestine and fixed with a membrane that degrades in the body, using the patient’s tissue to repair the mucosa. The patient has been reported to recover well following treatment.

### Other organoids

Mammary organoids hold promise for breast remodeling. A novel bioprinting system was developed by Reid et al. [234] to produce human mammary organoids by precisely controlling the spatial distribution of each cell through microneedle insertion of bioink into a collagen matrix. The authors highlighted bioprinting as a promising avenue for generating personalized tissue implants for breast reconstruction. Mollica et al. [235] used a 3D bioprinter to implant human mammary epithelial cell lines into various types of ECM and formed different types of tissue structures. The complex structure of the mammary gland hinders mammary regeneration. No studies have been conducted to transplant mammary organoids into animal models or humans or perform normal physiological functions.

To revolutionize mammary prosthesis therapy and regenerate human mammary organoids to approximate the structure and function of the mammary gland, a great deal of research remains warranted.

Lung organoids are promising for the treatment of airway diseases such as chronic obstructive pulmonary disease (COPD). Weiner et al. [236] developed an alveolar type 2 (AT2) organoid and transplanted it into mice infected with influenza. The transplanted AT2 organoids showed good implantation in vivo. However, these organoids did not improve the oxygen exchange capacity of infected recipient mice. This suggests that lung organoids in the body are not performing their physiological functions as expected.

The production of transplantable kidney organoids is extremely important as several patients with end-stage renal disease rely on conventional renal replacement therapy. As one of the first attempts to transplant kidney organoids, Xinaris et al. [237] implanted renal-like organoids beneath the renal capsule of male thymus-free nude mice and observed the formation of vascularized glomeruli with fully differentiated capillary walls, performance of relevant physiological functions (glomerular filtration and tubular reabsorption), and maturation of erythropoietin-producing cells. Kidney organoids derived from mouse embryonic kidneys were implanted under the renal capsule of immunodeficient mice. The results showed reestablishment of the arterial network and glomerular formation [238]. In the absence of any exogenous vascular endothelial growth factor, renal-like organoids produce host-derived vascularization.

Transplanted kidney organoids exhibit functional glomerular perfusion, glomerular vascularization, and maturation of podocytes [239]. Although previous studies have explored the regenerative medicine applications of kidney organoids, the necessary physiological functions (e.g., urine production and blood flow) have not been measured in animal models. Further studies are warranted to support the clinical translation of kidney organoids.

A healthy heart transplant is the cure for patients with congenital heart disease. Varzideh et al. [240] developed the first hiPSC-derived cardiac organoid for transplantation, induced structural organization of myogenic fibers, and enhanced gene expression by stimulating contractile coupling through in vivo transplantation. Four weeks later, the organoids showed expression of myocardial markers, electrophysiological activity, highly organized sarcomeric structures, and extensive neovascularization. Lee et al. [241] constructed iPSC-derived self-organized heart organoids (HOs) and verified the simulation of ventricular, epicardial/myocardial, and atrial/ventricular regions. After transplantation, HOs remain functional through the vascularization. Due to the complexity and diversity of the cardiac system, determining how to construct cardiac organoids that can perform pump function after transplantation remains challenging. Since iPSC-derived cardiomyocytes retain their embryonic properties even after in vitro differentiation, improving cell maturation is another challenge to be addressed.

## Constraints and Challenges: Achieving Clinical Translation

Despite advances in regenerative medicine made using engineered organoids, several challenges and limitations in clinical translation remain, which are discussed in this section, along with potential solutions.

### Culture

Due to the special culture conditions of organoids, automated platforms cannot be organically integrated with them, which significantly reduces the efficiency of organoid culture. However, the creation of robotics and artificial intelligence can address this challenge [122]. Alternative culture systems (e.g., rotating bioreactors) can overcome barriers to large-scale fabrication of organoids [99,122,242]. In addition, Matrigel is frequently found in organoid culture environments. Despite its several advantages, potential risks due to mouse contaminants, including the potential for tumorigenesis and immune responses, remain. These problems are further exacerbated by high variability. Attempts have been made to develop fully defined biomaterial-based hydrogels to overcome these obstacles and meet good specifications [153,243]. ECM macromolecule-based hydrogels can better support cell differentiation and are highly available and cost-effective [244].

However, hydrogels with monotonous compositions lack several natural components of the ECM and cannot provide the microenvironment required for the complete differentiation of organoids. In contrast, hydrogels derived from decellularized tissues retain the microenvironmental state of natural tissues and are more conducive to supporting the growth of 3D organoids [245,246]. However, dECM hydrogels have not been fully defined and may have donor-specific limitations. This further hinders the adaptation of the composition of hydrogels to meet experimental needs and further studies. Hydrogels based on synthetic polymers allow for better control of the mechanical properties of gels and are not limited to ECM. Researchers have developed “hybrid gels” that combine the advantages of synthetic gels and biogels, incorporating partial microenvironmental signals into PEG-based hydrogels to support iPSC differentiation [247]. However, these hybrid gels show limited cellular function, indicating the absence of key biochemical cues or growth factors. Further studies to determine the appropriate metrics are required for optimal combination, making the development of such hybrid gels both time-consuming and costly. Advances in hydrogel functionalization methods have provided more options; however, Matrigel may still be a good solution for the organoid culture [64]. Future research will help facilitate the translation of organoids grown in Matrigel for clinical applications, following thorough analysis.

Microorganisms play key roles in human physiological and pathological activities [248]. Currently, microbiota and organoid coculture techniques offer new possibilities for the treatment of diseases. Microinjections into the endocavity of organoids promote interactions with microorganisms at the epithelial level of the organoid and produce less cytotoxicity, which is a more desirable method of microbial delivery [249].

Compared with the external environment, organoids contain a lower concentration of oxygen; therefore, this method can help anaerobic microorganisms to reduce their exposure to oxygen. Microdevices have been developed for reproducible and efficient microinjection of microorganisms into the inner lumen of organoids [250]. To address the limitations of traditional intra-organoid microinjections, microorganisms have been suspended outside organoids and formed the tip of organoids (the epithelium rather than the basal lamina) on the outside to facilitate contact with the microorganisms. However, the instability of the organoid’s structural internal and external flip-flops remains to be addressed [251]. The use of monolayer cultures of organoid origin is another potential strategy that can facilitate access to samples and favors microbial entry [252]. However, this is detrimental to realizing organoid functional integrity and may have limited application in regenerative medicine. Significant progress can be made in developing the coculture paradigm more permanently [253].

### Function

The current shortcomings of organoids (e.g., functional disability, limited access to analysis, and variability) have hindered the translation of organoid technology into regenerative medicine. Addressing these issues will provide novel insights.

#### Limited maturity

Several organoids exhibit impressive physiological functions corresponding to their original organs. However, no established organoid system replicates the full function of an organ. Researchers have attempted to coculture stem cells to create organoids that reproduce all the functions of the original organ. However, intercellular interference makes it difficult to control the direction of differentiation by directly coculturing different types of stem cells. Today, it is possible to construct bionic hierarchical structures reflecting the spatiotemporal characteristics of organ development using integrating bottom-up fabrication techniques or emerging 3D printing technologies, creating the possibility of constructing fully functional mature organoids. For example, Kupfer et al. [254] used technological advances in 3D bioprinting to achieve, for the first time, macroscopic-scale beating function in a geometrically complex, perfusable chamber structure, completing the unification of cardiac conduction and pumping functions, and taking a substantial step toward a macroscopic chamber model of the human heart. This approach can also be applied to many other cell types that are less able to migrate and proliferate following differentiation, providing an important reference for the construction of fully mature organoids. Furthermore, Shahriyari et al. [255] added growth factors in a strictly sequential manner, directed differentiation of hPSC into embryonic muscle progenitor cells, and converted fetal muscle into 3D muscle, which led to the engineering of skeletal muscle (ESM) with realistic and advanced structural and functional properties.

#### Absence of key structures

Organoids lack key specialized cell types, resulting in a lack of vasculature or mesenchyme, preventing homogeneity with the native organ. This is related to the current limited understanding of stem cells and the poor generalization of widely used biosignatures. In contrast, the lack of consistent cellular organization in organoids that establish multiple mesenchymal compartments creates an obstacle to transplantation as well as the growth of organoids. Bone tissue engineering suggests that stem cells can be cocultured with vascular endothelial cells to construct vascularized organoids. Growth factors (e.g., vascular endothelial growth factor) promote the generation of vascular networks [256]. Bertassoni et al. [257] printed engineered constructs along with the surrounding tissue and performed external perfusion of these constructs. The authors reported a hydrogel-built vascularization technique that confirmed endothelial monolayer formation within the fabrication channel.

Noor et al. [258] used personalized hydrogels as bioinks to 3D print complete, thick vascularized tissue in just one step. When organoids are transplanted into the host, the process of vascularization mimics the angiogenesis in vivo. In vivo, vascularization is more effective in producing fully functional organoids and more conducive to organoid survival [259]. Koning et al. [260] transplanted kidney organoids into the renal capsule of mice. The host’s vascular network invaded the organoid and actively connected with the organoid vasculature. Using a transplantation approach, the authors described the vascularization of renal organoids.

Similarly, Takebe et al. [85] cultured vascularized human liver organoids. Umbilical vein endothelial cells, iPSC-derived liver endodermal cells, and MSCs self-organized into liver buds. Organoid chips may be an effective vascularization solution. The progress made in organoid vascularization will provide an important reference for organoids to reproduce the key structures of the original organ and thus achieve the restoration of full physiological functions.

#### Limited lifespan

Enlarged organoids reduce the diffusion efficiency of nutrients and metabolic wastes, thereby inhibiting further organoid growth or even causing death. Placing organoids in bioreactors or microfluidic chips may reproduce the biomechanical relationships between organs, favoring their long-term survival. To apply organoids in regenerative medicine, they must establish normal connections with the host organs in vivo. A novel microfluidic multiorgan system generalizing the human hepatopancreatic islet axis has been previously investigated [261]. Cocultured liver and islet organoids grow well and show specific organ functions. In a recent study, a sliced neocortical organoid (SNO) system was produced. The SNO system involves precisely slicing the forebrain organoid into a disk shape, exposing the progenitor cell area to the external culture environment [262]. Cell proliferation and neurogenesis persist in long-term SNO cultures, while the scale of cellular layers (e.g., neuronal and progenitor layers) is much larger than that in conventional methods.

This innovative approach overcomes the problems of hypoxia and internal cell death in organoid culture, extends the life span of organoids, and opens the possibility of constructing larger and more fully functional organoids.

#### Heterogeneity

Organoids are heterogeneous in terms of cellular composition, size, and shape due to the uncertainty of their intrinsic cell fates. This heterogeneity is influenced by several factors, such as laboratory conditions and the type or source of stem cells. The variability in stem cell lines and differences in genetic background may cause misinterpretation of results; therefore, replications across multiple cell lines may be necessary for practical validation. It is best to use multiple biological replicates or homologous controls for organoids to ensure reproducibility and consistency. Organoids, like other models, have flaws and limitations. Researchers should not overinterpret findings as basic facts, especially in the absence of validated results, but should use these methods with a clear understanding of their limitations [263]. Therefore, reducing heterogeneity is crucial to realizing the potential of organoids in regenerative medicine. Novel gene editing methods (e.g., CRISPR/Cas9) allow for the precise gene editing of organoids to directly obtain target constructs. Standardization and automation of steps and modalities of culturing organoids can improve reproducibility and reduce variability. For example, Ao et al. [264] developed a one-stop microfluidic platform that provides a simple, scalable, and reproducible method for culturing human brain organoids, which can be used for other human organoids (e.g., retina, liver, and kidney).

The microfluidic platform combines a one-stop solution, a gas–liquid interface, and a perfusable culture chamber compared with traditional manufacturing methods, simplifies the fabrication process, and allows for the production of large numbers of organoids (169 organoids per 3.5 cm × 3.5 cm device area) without fusion.

### Personalized healthcare

Most clinically employed organoids are used in disease modeling [265] and drug studies [266]. Few studies have focused on the application of organoids in tissue repair; however, they do not provide specific information regarding the timing and potential costs associated with delivering personalized organoids to patients. These factors could significantly impact the future adoption of engineered organoids in healthcare.

The use of customized organoids for clinical treatment may not be applicable in all cases for several reasons. First, the lengthy process of organoid culture may be impractical for organ injuries requiring emergency care. Second, the patient-derived autologous primary organoid may still be affected by the disease. Third, due to the limitations of current culture techniques, in some cases, the structure and function of cultured organoids may not be identical to those of the original organ. However, transplantation of nonautologous organoids can be achieved through human leukocyte antigen (HLA) knockout [267] or PD-L1 overexpression [268], thereby reducing the waiting time for patients. To achieve donor–recipient matching, organoid biobanks can be created using immunocompatibility methods similar to those used in liver transplantation.

If the organoid is derived from the patient, posttransplantation immune rejection is not a factor to be considered, and immune cells will not be activated and impede growth. Organoids successfully integrate with the host’s original cells and microenvironment (e.g., neural organoids enable connections between injured axons, bone organoids enable bone healing of fractured ends, and skin organoids can cover injured skin and grow hair) because their cellular composition and properties are highly similar. Organoid and normal host cells can perform various life activities and physiological functions in a coordinated manner and can treat certain diseases (e.g., scleroderma [187]) by altering the local abnormal pathological microenvironment. However, many preclinical studies have been done in mice, and to achieve integration with organoids, immunocompromised mice must be artificially cultured to prevent host cells from recognizing and destroying the organoids. Researchers are overcoming the problems of allogeneic organoid transplantation and observing how organoids achieve integration with the organism. The allogeneic cartilage organoid constructed by Abe et al. [198] did not elicit immune exclusion in primates. Integration of the organoid with natural cartilage (using staining) was observed at 17 weeks after transplantation. At the same time, no accumulation of immune cells (including CD3^+^ T lymphocytes) was observed in the transplanted group, indicating that the allogeneic organoid did not elicit an immune response for at least 4 months. More efficient and advanced engineering techniques remain warranted to construct autologous or allogeneic organoids, thus avoiding the long-term or lifelong use of immunosuppressants (as is the case with organ transplants).

For the marketization of organoids for regenerative medicine, considering the costs borne by patients is important. Relevant data-based studies have shown that the cost for patients for in vitro donor liver repair is estimated to be between €378,000 and €903,000. The cost of organoid-based cell therapy (CT) is estimated to be between €276,000 and €801,000 (since CT requires a smaller number of cells, which significantly reduces the cost of tissue production; however, not all patients meet the indications for CT). Alarmingly, the cost of organoid-based tissue engineering is projected to be €6.858 million to €7.041 million [37]. Preliminary cost estimates suggest high costs associated with organizing production [269]. Costs may vary over time; as technology evolves, cost advantages will emerge due to economies of scale and incremental innovation. The total cost of sequencing the human genome has gone from €3 billion to just €1000 [270].

Attempts have been made to reduce costs by optimizing protocols for cost-effective cultures, considering the economic and accessibility issues for clinical patients. Some researchers have designed simple miniaturized dialysis culture systems, thus validating the feasibility of cost-effective high-density liver differentiation using minimal growth factors [271]. This will provide conceptual support for all organoids in reducing costs for clinical applications. In addition, although the cost of organoids for regenerative medicine translation has not been considered, previous studies have explored more efficient and cost-effective strategies for culturing organoids. A low-cost strategy was used to propagate intestinal organoids [272]. The cost and efficiency of tubal organoids cultured in different media have also been assessed [273].

### Ethical questions

Organoids are grown from human cells, which raises several ethical questions. New ethical analyses are therefore needed in all aspects of organoids. First, in the use of ESCs to create organoids, the embryo must be destroyed to obtain ESCs, which raises ethical concerns [92]. Second, data on the risks associated with some emerging technologies remain lacking, and comprehensive regulations are required to monitor specialized practices in clinical trials to prevent the emergence of new biological risks. The purpose of monitoring is to address potential risks without limiting organoid research. In addition, some studies might have exaggerated the results of certain organoid studies, which could potentially influence the public perception of organoids and thus generate additional controversy [274]. Finally, ethical research should also be extended to areas where clinical applications are expected, such as organoid transplantation and its impact on patients, healthcare institutions, and society. Therefore, policies must be developed to protect patients undergoing tissue repair and biofunctional reconstruction using organoids, as well as the healthcare professionals involved [263].

## Conclusions and Outlook

Organoids can reproduce the 3D structure and function of organs and hold great promise for clinical applications. The current challenges that remain to be addressed include keeping pace with the rapid evolution of biotechnology, translating organoid-related concepts and tools into real organ therapeutic strategies, making full use of bioengineering tools to cultivate organoids, and accomplishing patient-specific personalized tissue repair and biofunctional reconstruction using more advanced engineered organoids.

In conclusion, organoids hold immense potential for tissue repair and biofunctional reconstruction in almost all human organs. As engineering organoid technology continues to advance, it will undoubtedly usher in a new era in the field of human regenerative medicine. With technological advancements, the use of engineered organoids for complete, customizable, and efficient tissue regeneration and biofunctional reconstruction may become as easy as “pulling a cell phone out of your pocket.”

## Data Availability

Data availability is not applicable to this article as no new data were created or analyzed in this study.
