# Supplementary material for: Advancing Organoid Engineering for Tissue Regeneration and Biofunctional Reconstruction
Source: Biomater Res. 2024 Apr 15;28:0016. doi: 10.34133/bmr.0016 (PMC11018530; doi:10.34133/bmr.0016)
Supplement: Supplementary 1 — Figs. S1 [file bmr.0016.f1.docx]

Supporting Information

**Advancing Organoid Engineering for Tissue Regeneration and Bio-functional Reconstruction**

**Authors**

Hairong Jin^1,2,3†^, Zengqi Xue^2†*^, Jinnv Liu^2^, Binbin Ma^4^, Jianfeng Yang^1,2^, Lanjie Lei^1*^

**Affiliations**

^1^Institute of Translational Medicine, Zhejiang Shuren University, Hangzhou, 310015, China.

^2^The Third Affiliated Hospital of Wenzhou Medical University, Wenzhou, 325200, China.

^3^Ningxia Medical University, Ningxia, 750004, China.

^4^Department of Biology, The Johns Hopkins University, Baltimore, MD 21218, USA.

†These authors contributed equally to this work.

**^*^Address correspondence to:** leilanjie1988@163.com(L.L.); xuezengqi@wmu.edu.cn(Z.X.)


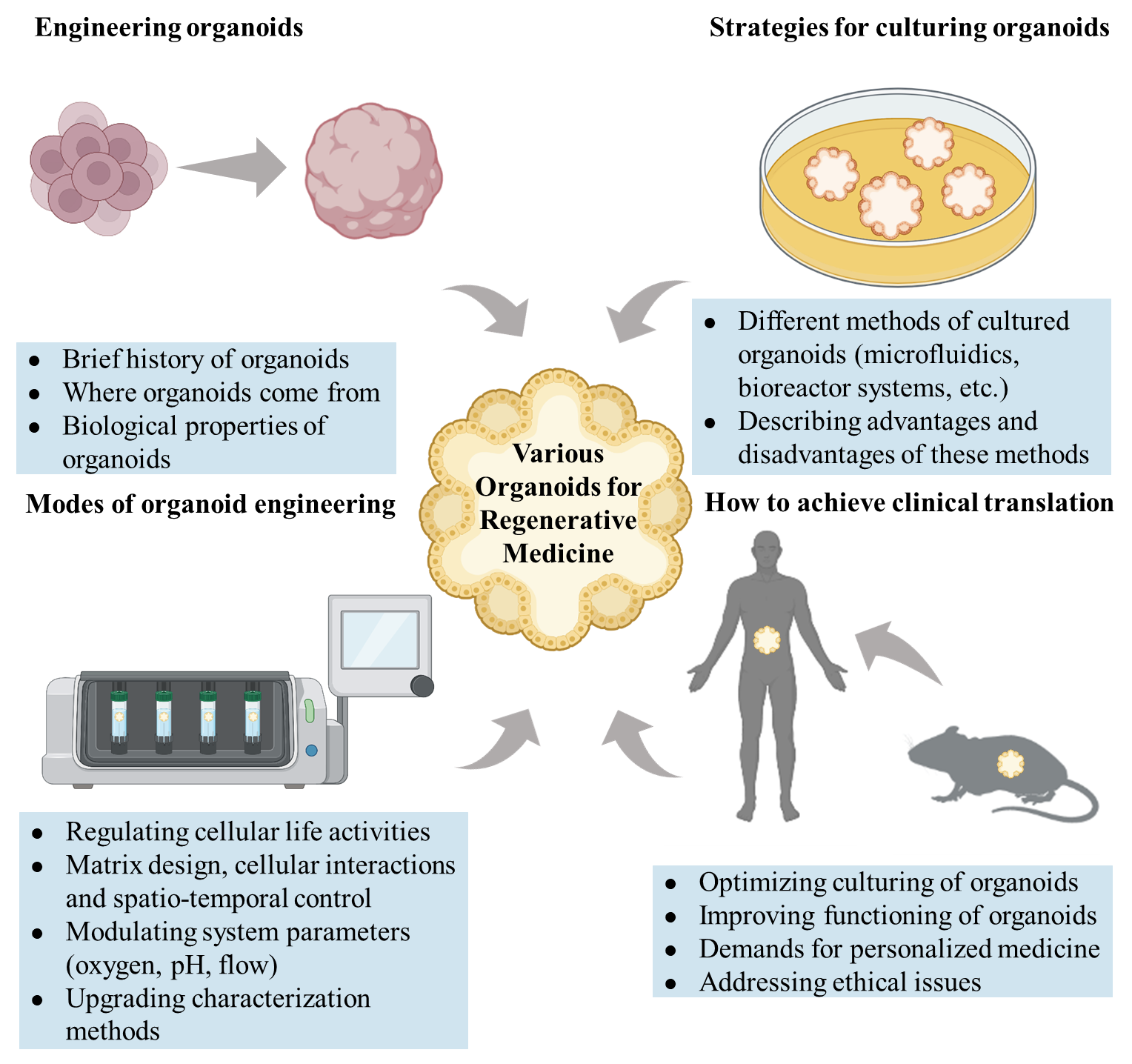


**Fig.S1.** Schematic illustrations of advancing organoid engineering for tissue regeneration and bio-functional reconstruction. Created with BioRender.com. (Agreement number: OK26GU1NV4).
